# Robust leaf trait relationships across species under global environmental changes

**DOI:** 10.1038/s41467-020-16839-9

**Published:** 2020-06-12

**Authors:** Erqian Cui, Ensheng Weng, Enrong Yan, Jianyang Xia

**Affiliations:** 1 Zhejiang Tiantong Forest Ecosystem National Observation and Research Station, State Key Laboratory of Estuarine and Coastal Research, Shanghai Key Lab for Urban Ecological Processes and Eco-Restoration, School of Ecological and Environmental Sciences, East China Normal University, Shanghai, 200241 China; 2 Research Center for Global Change and Ecological Forecasting, East China Normal University, Shanghai, 200241 China; 3 Center for Climate Systems Research, Columbia University, New York, NY 10025 USA; 4 Forest Ecosystem Research and Observation Station in Putuo Island, East China Normal University, Shanghai, 200241 China; 5 Institute of Eco-Chongming (IEC), Shanghai, China

**Keywords:** Plant ecology, Climate-change ecology

## Abstract

Recent studies show coordinated relationships between plant leaf traits and their capacity to predict ecosystem functions. However, how leaf traits will change within species and whether interspecific trait relationships will shift under future environmental changes both remain unclear. Here, we examine the bivariate correlations between leaf economic traits of 515 species in 210 experiments which mimic climate warming, drought, elevated CO_2_, and nitrogen deposition. We find divergent directions of changes in trait-pairs between species, and the directions mostly do not follow the interspecific trait relationships. However, the slopes in the logarithmic transformed interspecific trait relationships hold stable under environmental changes, while only their elevations vary. The elevation changes of trait relationship are mainly driven by asymmetrically interspecific responses contrary to the direction of the leaf economic spectrum. These findings suggest robust interspecific trait relationships under global changes, and call for linking within-species responses to interspecific coordination of plant traits.

## Abstract (web summary)

It is unclear whether rapid global change will lead to unexpected trait combinations. In this global meta-analysis on vascular plants, Cui et al. show that, although within-species responses do not always follow the leaf economic spectrum, the slopes of interspecific trait relationships are robust to rapid environmental change.

## Introduction

Leaf traits represent plant functional strategies and have fundamental effects on vegetation properties and ecosystem functions^1–3^. Divergent leaf traits between species present strong trade-offs that called the leaf economic spectrum (LES)^2^. For example, plants with low specific leaf area (SLA) and leaf nitrogen content (N_m_) have slow photosynthetic returns, while plants with the opposite traits have fast photosynthetic returns^4–6^. Large trait variations between species typically represent the evolutionary divergences resulting from genotypes or species turnover. The plant LES integrates trade-offs of leaf traits and thus provides a useful framework for elucidating leaf-to-ecosystem scaling and for modeling vegetation functional diversity and dynamics in a changing climate^7–10^. In addition, correlations among leaf traits can provide significant constraints on the estimates of vegetation–atmosphere carbon exchange^11–13^.

Leaf traits of a given species can also vary widely in response to environmental changes through a diverse array of physiological, behavioral, and ecological mechanisms^14,15^, which is defined as leaf trait plasticity^16^. Understanding leaf trait plasticity is a major challenge for predicting plant responses to global environmental changes^17,18^. Various empirical trait–environment relationships^9,19,20^ and trait–trait correlations^21,22^ have been presented to incorporate trait variations into mechanistic vegetation models. However, the extant patterns are the results of historical evolutionary selections from their specific environmental conditions^23^. Leaf trait plasticity may differ in magnitude and even direction from the existing trait–environment relationships^24,25^. Thus, it is still unclear whether the plasticity-caused trait changes would follow the principles derived from current LES analyses. In fact, the uncertain changes of trait-pairs in response to environmental changes have long been limiting the predictive application of the interspecific trait relationships^26^. Furthermore, the projected novel climate may lead to nonanalog plant functional types and/or trait combinations^27–29^. A better understanding of how leaf traits and their relationships in response to future environmental changes is crucial for predicting vegetation distribution and ecosystem function^30^.

**Fig. 1:**
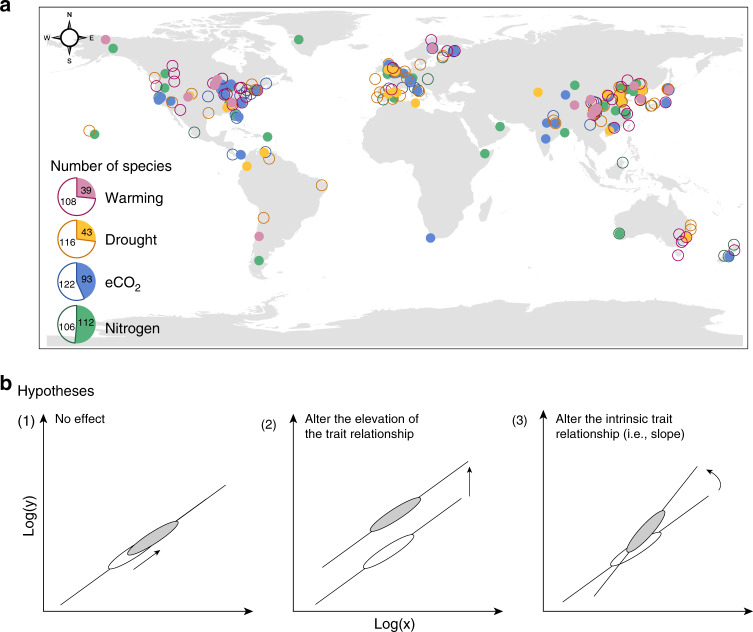
Distribution of the global trait database and the hypotheses in this study. **a** Locations of the study sites in this dataset. The filled circles represent the distribution of field experiments and the open circles for the locations of environmentally controlled experiments. The inset shows the number of species for environmentally controlled and field experiments. Note that locations of the environmentally controlled experiments are determined by latitude and longitude of the experimental sites. **b** Hypotheses depicting possible effects of global environmental changes on log–log trait relationships (of the form ${\mathrm{log}}y = k\, \times \,{\mathrm{log}}\,x + b$). Here, we assumed that the trait relationships remain constant (1), the scaling exponents of the trait relationships remain unchanged but the elevation vary (2), and/or the interspecific trait relationships vary (3) under global environmental changes. The arrows indicate the directions of trait plasticity.

With the advent of global change manipulative experiments which characterize the effects of environmental changes on leaf traits, directly quantifying leaf trait plasticity is possible^31,32^. Here, we combine leaf traits data from 102 field experiments and 108 environmentally controlled experiments (Supplementary Table 1 and Fig. 1a). The field experiments in this database cover extensive climatic regions, with mean annual temperature (MAT) ranging from −9 to 27 °C, and precipitation from 40 to 4158 mm (Supplementary Fig. 1). The environmental temperature of environmentally controlled experiments ranges from 5 to 33 °C and relative humidity from 27 to 97%. In total, we compile records of 515 species from 90 families, 60% of which from environmentally controlled experiments and 40% from field experiments (Inset in Fig. 1a). Based on these data, we quantify the plasticity of net photosynthetic rate (A_m_), N_m_, and SLA to multiple global environmental changes. In addition, we analyze if the changes in trait-pairs tend to be follow the directions of the interspecific trait relationships. Furthermore, we test whether the intrinsically interspecific trait relationships hold under future environmental changes. Leaf traits show allometric relationships with the form of power function:^1,2^ $y = cx^k$. In the power law, *c* is the coefficient and *k* the dimensionless scaling exponent^33^. On the logarithmic axes of trait relationships $\left( {{\mathrm{log}} \,y = k \, {\mathrm{log}} \,x + b;\,b \equiv {\mathrm{log}} \, c} \right)$, slope (*k*) represents the fundamental mechanisms of the bivariate relationship and slope elevation (intercept, *b*) indicates the resource-use efficiency^34^. As illustrated in Fig. 1b, if both the slope and elevation of the log–log axes have no response to environmental changes, then the empirical interspecific trait relationship stays unchanged. Alternatively, if the slope keeps constant but the elevation shifts, then the scaling exponent remains robust but the resource-use efficiency varies. Conversely, if the slope changes significantly, then the empirical trait relationships are shifted. In this study we show that, although the direction of trait change in response to environmental changes varies enormously between species and generally does not follow the LES, the slopes of interspecific trait relationships hold stable.

## Results

### Response of leaf traits to global environmental changes

**Fig. 2:**
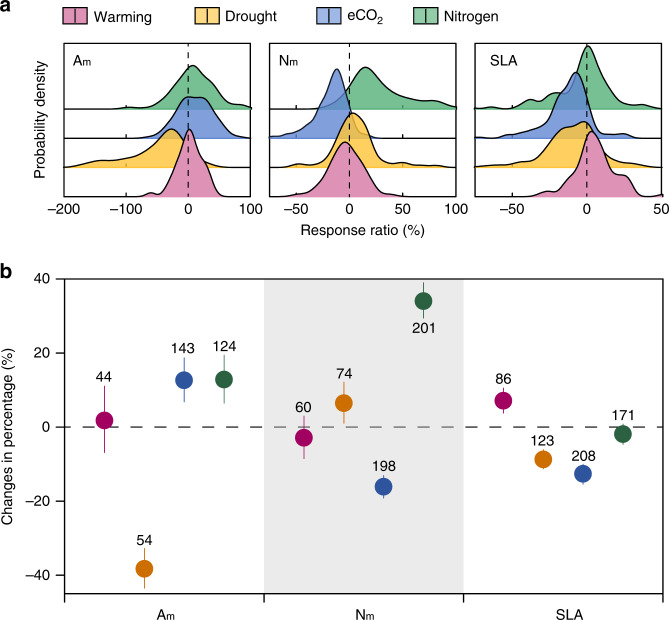
Percentage changes of leaf functional traits under global environmental factors. **a** Probability density of response ratios of leaf traits to warming, drought, eCO_2_, and nitrogen addition. **b** Effects of global environmental changes on leaf functional traits. Circles represent the global mean changes, and error bars are 95% credible intervals on the mean. The horizontal dashed line indicates 0 (no effect). The number of species under treatments of warming, drought, eCO_2_, and nitrogen addition are 44, 54, 143, and 124 for A_m_, 60, 74, 198, and 201 for N_m_, and 86, 123, 208, and 171 for SLA (i.e., the numbers shown near the bars).

The response ratios of leaf traits to global environmental changes were normally distributed (Fig. 2a). On average, experimental warming had no significant effect on A_m_ and N_m_, except for a slightly increase in SLA values by 6.9% (Fig. 2b). In contrast, all trait values varied significantly when the plants experience drought condition. Experimental drought significantly decreased A_m_ (−38.3%) and SLA (−8.7%), respectively, but increased N_m_ (6.5%). The elevated atmospheric CO_2_ concentration roughly induced 16.1% and 12.6% reductions in N_m_ and SLA but increased A_m_ by 12.6%. Nitrogen addition significantly increased N_m_ (34.0%) and A_m_ (12.8%), respectively. However, the commonly measured morphological trait SLA did not shift significantly under nitrogen addition.

Plant growth environment (field or environmentally controlled) and treatment conditions (treatment strength and duration) were important factors affecting trait response^35^. Our results showed that field experiments presented larger effect on leaf photosynthesis, but lower impact on leaf nitrogen and SLA than environmentally controlled experiments (Supplementary Fig. 2). Furthermore, the responses of some leaf traits were affected by treatment strength of warming, drought, and nitrogen addition as well as the experimental duration of elevated CO_2_ (Supplementary Table 2, Supplementary Fig. 3). The percentage changes in A_m_ was negatively correlated with warming strength and temperature conditions, and the warming effects varied from positive to negative with the increase of the temperature (Supplementary Fig. 3a). The effect of drought on A_m_ increased linearly with enhanced drought strength (*R*^2^ = 0.21, *P* < 0.01). Similarly, the positive responses of leaf nitrogen to nitrogen addition increased linearly with applied dose (*R*^2^ = 0.14, *P* < 0.01). Our results also revealed that response of SLA to eCO_2_ logarithmically decreased with increasing experimental duration.

### The direction of changes in pairwise traits under global environmental changes

**Fig. 3:**
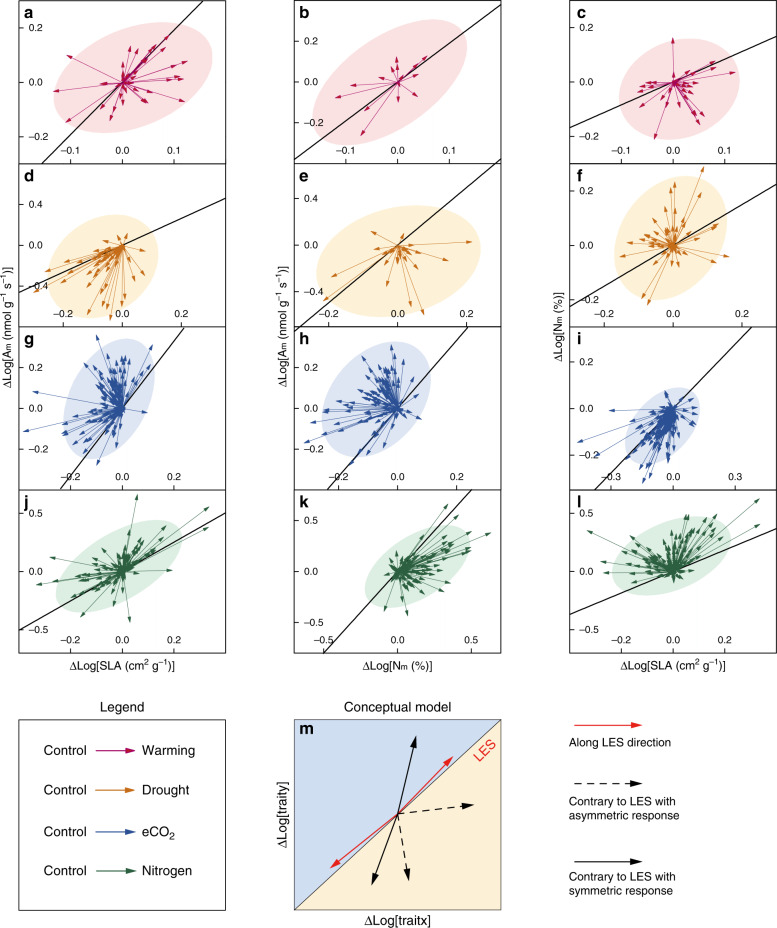
Plasticity of species-level pairwise traits under global environmental changes. **a**–**l** Plasticity of species-level pairwise traits under global environmental changes (Supplementary Note 2). Each colored arrow represents the direction of one species. The bold black line represents the interspecific leaf trait relationship. The colored ellipses are 95% confidence level of the scatters. **m** Conceptual model depicting three possible directions of pairwise traits: follow the LES direction (red arrows); contrary to the LES direction with asymmetric response (black dashed arrows); contrary to the LES direction with symmetric response (black solid line). The black solid line represents the LES direction. The arrows represent the major directions of the pairwise traits.

Importantly, we also tested the consistency between direction of trait plasticity and the interspecific trait relationships. We plotted the direction of trait plasticity induced by environmental changes for each species into trait–trait space, and found that the changes in trait-pairs were divergent between species. In addition, we found that only the plasticity of N_m_–SLA relationship under eCO_2_ and the plasticity of A_m_–SLA under nitrogen addition tended to be follow the LES direction (Fig. 3i, j). Most of the trait plasticity was equally abundant in all directions of the trait–trait space under warming and drought (Fig. 3a–f). Furthermore, the directions of trait plasticity under eCO_2_ and nitrogen addition were mainly asymmetric distributed on one side of the LES (Fig. 3g–l).

### Plasticity of the interspecific trait relationships under global environmental changes

**Fig. 4:**
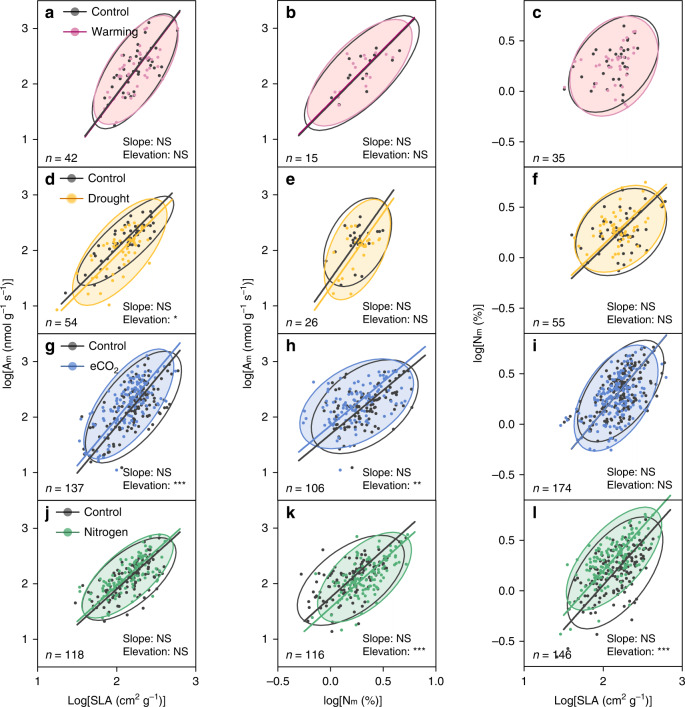
Robustness of leaf trait relationships under different global environmental changes. The panels show the effects of warming (**a**–**c**), drought (**d**–**f**), eCO_2_ (**g**–**i**) and nitrogen addition (**j**–**l**), respectively. The bold lines represent SMA regressions of leaf trait relationships. The ellipses show the 95% confidence level of the original scatter. The homogeneity among SMA slopes via a permutation test and for differences in SMA elevation via the SMA analog of standard ANCOVA. The statistics information is shown in Supplementary Table 3. The number of species under treatments of warming, drought, eCO_2_, and nitrogen addition are 42, 54, 137, and 118 for A_m_–SLA, 15, 26, 106, and 116 for N_m_–SLA, and 35, 55, 174, and 146 for N_m_–SLA (i.e., the numbers shown near the ellipses). The Significance of changes in slopes and elevations: NS: *P* > 0.05; *P < 0.05; **P < 0.01; ***P < 0.001.

**Fig. 5:**
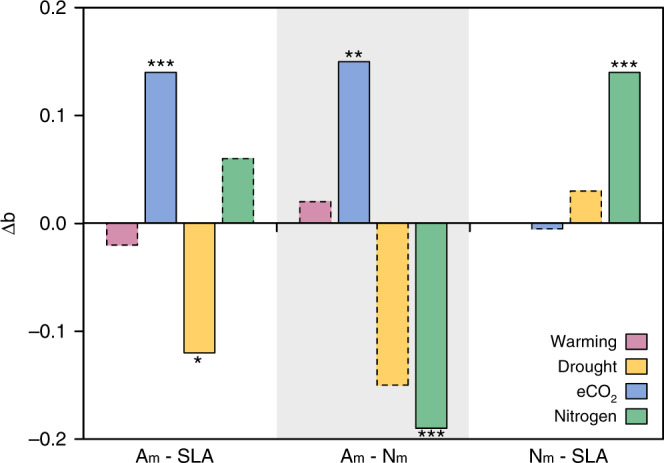
Changes of elevations (*b*) for trait relationships under global environmental changes. The differences in SMA elevation via the SMA analog of standard ANCOVA. Significance: dashed borders: *P* > 0.05; *P < 0.05; **P < 0.01; ***P < 0.001.

As shown by the standardized major axis (SMA) regression, the SMA slopes of the interspecific trait relationships held stable under global environmental changes, but only the SMA elevations varied. Experimental warming had no significant effect on the elevations of interspecific trait relationships (Fig. 4a–c and Supplementary Table 3). The elevation for A_m_–SLA correlation was slightly altered by drought (Fig. 4d and Supplementary Table 3). The relatively lower elevation revealed a reduction in photosynthetic rate at a given unit of SLA under the drought treatment. Similarly, the increased CO_2_ treatment altered the A_m_–N_m_ and A_m_–SLA relationships via significant enhancement on their elevations (Fig. 4g, h and Supplementary Table 3). The increased elevations respectively indicated higher photosynthetic rate for a given leaf nitrogen concentration and higher photosynthetic rate for the equivalent leaf area. Meanwhile, the nitrogen addition significantly lowered the elevations of A_m_–N_m_ correlation, but lifted the elevation of SLA–N_m_ relationship (Fig. 4k, l and Supplementary Table 3). Overall, the elevation of A_m_–N_m_ correlation was most sensitive to global environmental changes, with the elevation changes were −0.19 and 0.15 under nitrogen addition and elevated CO_2_, respectively, (Fig. 5). The elevation of N_m_–SLA relationship was relatively robust, only significantly altered by nitrogen addition. Note that the relationship change of SLA–N_m_ under warming was not detected because of their nonsignificant correlation (Supplementary Table 3).

Recent studies have emphasized the equivalent importance of mass- and area-based interspecific trait relationships^36,37^. Here, we also tested the robustness of area-based interspecific trait relationships under environmental changes. First, we reconfirmed the disparate patterns of mass- and area-based trait relationships^3,37^ (Supplementary Fig. 4). The area-based A–SLA and A–N were weakly related, whereas stronger correlations for mass-based traits were presented. In addition, contrasting relationships were found between the area- and mass-based N–SLA. Then we showed that the slopes of area-based N_a_–SLA relationships remained unchanged (all *P* > 0.05, Supplementary Table 4). In addition, we found different elevation changes for mass- and area-based N–SLA relationship under elevated CO_2_ (Supplementary Fig. 4o, r). The robust N_m_–SLA correlation under eCO_2_ resulted from the proportional changes of N_m_ and SLA along the original axis, while larger eCO_2_-dependent decrease in SLA than N_a_ was responsible for the significant reduction in elevation of N_a_–SLA.

It has been confirmed that species from different functional groups and growth environment (field and environmentally controlled) may have different eco-physiological constraints when experiencing global environmental changes^38,39^. We found that interspecific trait relationships have consistent slopes between angiosperm and gymnosperm woody species as well as between dicotyledons and monocotyledons were consistent. Only the slopes of the N_m_–SLA relationship were different between C_3_ and C_4_ herbs (Supplementary Table 5 and Supplementary Fig. 5). Moreover, the slope of trait relationship was not significantly affected by environmental factors in any of the functional groups (all *P* > 0.05, Supplementary Tables 6–8). The environmental changes altered the elevation of trait relationship in angiosperm woody but not gymnosperm woody species (Supplementary Fig. 6a). Dicotyledons and monocotyledons showed similar trait adjustment strategies under eCO_2_ and nitrogen addition (Supplementary Fig. 6b). We also detected a lower sensitivity of the photosynthetic nitrogen use efficiency in C_4_ than C_3_ plants to eCO_2_ (Supplementary Fig. 6c). Overall, different response among functional groups have implications for how these plant groups are likely to perform in future climate change. In addition, we found that the slopes of trait relationships from field and environmentally controlled experiments were both unchanged (all *P* > 0.05, Supplementary Table 9), and their elevations changed in the same direction (Supplementary Fig. 7a). The impact of treatment strength on the elevation shift of trait relationship was only detected in A_m_–SLA under drought (Supplementary Table 10 and Supplementary Fig. 7b).

## Discussion

This study shows the high plasticity of leaf economic traits under global environment changes. Plants generally have great plasticity in leaf characteristics to optimize their function under the prevailing changes in environmental conditions^40^. Globally, climate warming showed zero-sum impact on leaf traits (Fig. 2a). Our results corroborate the dependence of warming effects on temperature conditions^41^, and show that warming accelerate leaf photosynthesis in cold environments, but negatively affect leaf photosynthesis in warmer climates^35,42^ (Supplementary Fig. 3a). The negative effect of warming may result from warming-induced water deficit or the excessive temperature that beyond its optimum points^43^. In addition, both SLA and N_m_ of plants are sensitive to elevated CO_2_. The opposite responses of leaf photosynthesis and other leaf traits strongly suggest that leaf traits move in the opposite direction of the common recognized LES under the increased CO_2_ concentration^44,45^, as expressed by higher leaf photosynthesis and higher carbon investment in leaves. The reduced N_m_ under elevated CO_2_ has been attributed to the dilution effect due to rapid growth^46^ or redistribution of ecosystem N stocks^47^. Moreover, our results show that plants tend to alter N_m_ rather than SLA under higher nitrogen availability. SLA has been found to be a reliable indicator of plant resource-use strategy^48^. However, our findings corroborate a recent study^49^ that leaf nutrient, but not SLA, is an appropriate indicator of plant response to increased nutrients inputs. Considering the extremely high variability of leaf photosynthesis due to stomata closure under drought, A_m_ is not a reliable indicator of functional response under drought stress. As suggested by some previous studies^13,30^, the quantification of the leaf traits plasticity could also provide observational constraints on modeled vegetation and ecosystem function.

The unchanged slopes support the robustness of the reported trait relationships in disparate environmental conditions. We further propose a conceptual framework to show why the interspecific LES maintains despite the divergent trait plasticity (Fig. 3m). There are three response patterns of pairwise traits under environmental changes: (1) the direction of trait plasticity follows the LES; (2) the direction of trait plasticity is contrary to the LES with asymmetric responses; and (3) the direction of trait plasticity is contrary to the LES with symmetric responses. As shown by Fig. 3, most of the directions of trait plasticity were contrary to the LES with asymmetric responses, leading to the invariant slopes of trait–trait relationship under global environmental change. The observed elevation changes are mainly driven by the asymmetric responses which are contrary to the direction of LES (Fig. 3d, g, h, k, l). The across-species LES could shift when the trait plasticity is symmetric between upward and downward directions of the LES.

The interspecific trait relationships are mostly consistent across experimental types (field and environmentally controlled) and functional groups (Supplementary Note 1). However, the elevation of trait relationship varies in some cases. For example, the elevation changes are higher in the environmentally controlled than the field experiments (Supplementary Table 9), which might result from the faster growth rates of plants than that in the field^39^. The elevations of trait relationship show larger variations in angiosperms than gymnosperms (Supplementary Fig. 6a). This result suggests a more conserved trait adjustment strategy in gymnosperms than angiosperms, which has also been found in the root traits^50^. Due to the higher nitrogen use efficiency in C_4_ than C_3_ herbs^51^, it is expected that the elevation of A_m_–N_m_ relationship is greater in C_4_ than C_3_ herbs (Supplementary Fig. 6c). However, eCO_2_ treatment significantly increased the elevation of A_m_–N_m_ relationship for C_3_ but not C_4_ herbs. This finding suggests that the A_m_–N_m_ relationship could be more stable in C_4_ than C_3_ herbs under the higher atmospheric CO_2_ concentration. It should be noted that the sample size of our database is not large enough to compare all trait relationships between the control and treatment groups for all functional groups. However, the findings of some differences in the elevation of trait relationship between functional groups imply the need to explore trait variations across phylogenetically distant species. In fact, some recent global analyses have shown the importance of phylogenetic factors in explaining plant response to global changes^52,53^.

The elevation of A_m_–SLA correlation represents the photosynthetic efficiency per-unit leaf mass, which is affected by the investment in photosynthetic mass per-unit leaf area^54,55^. Similarly, the elevation of A_m_–N_m_ correlation indicates photosynthetic N-use efficiency, shaped by nitrogen allocation to Rubisco or Rubisco-use efficiency^56^. Plants are expected to increase investment in structural mass and therefore confer higher physical strength and greater resistance to drought stress^57^, which could decrease photosynthetic efficiency per-unit leaf area (Fig. 4e). Without evidence of significant increase in photosynthetic mass per-unit area or Rubisco nitrogen fraction^47,58^, the uplifted elevations for A_m_–SLA and A_m_–N_m_ correlations under eCO_2_ (Fig. 4h) could be attributed to the increased Rubisco-use efficiency. The reduced photosynthetic N-use efficiency under nitrogen addition has been reported due to the decreased fraction of nitrogen to Rubisco^59,60^ (Fig. 4j). In addition, the elevation changes in the N_m_–SLA correlation result from the variation of leaf nitrogen per-unit area, which is only significantly enhanced by nitrogen addition (Fig. 4l). Further researches are still needed for a deeper understanding of the physiological mechanism underlying the elevation change under future environmental changes.

The robust slopes of leaf trait relationship across species implie that the traits coordination could be used to predict ecological consequence of global environmental changes. In fact, plant traits are important parameters in regulating vegetation processes in the framework of Earth system models^11,61^. However, an increasing body of evidence has indicated the insufficient realism of current models for their ignorance of leaf traits variability and plasticity^28,62^. The widely demonstrated leaf traits plasticity can alter leaf structure and function, thus, plant productivity and land–atmosphere fluxes. Recent advances have incorporated leaf trait plasticity into models for a more realistic presentation of the responses of terrestrial ecosystem to climate change^25,30^. Currently, connecting trait relationships across plants, micro-organisms and animals remains a big challenge for ecology and biogeochemical modeling^63^. Our study suggests that scaling trait relationships from intra- to interspecies and even up to the community or ecosystem level^64^ is an important next step to implement trait-based approaches into modeling future dynamics of the Earth system.

In summary, the various patterns of the leaf trait relationships along climate gradient have long been limiting the predictive utility of such empirical correlations^15,34^. Such a weak predictive ability could largely stem from the divergent directions of the shifts in leaf traits under environmental changes among species. It is interesting that the divergent changes in pairwise traits have no impact on the slope of interspecific trait relationships at the global scale. Overall, this study indicates that the direction of changes in pairwise traits in response to global change varies enormously between species, and the within-species changes generally do not follow the direction of interspecific trait relationships. However, the slopes of interspecific trait relationships across species are stable under environmental changes, which indicate the fundamental mechanism of the bivariate relationship does not change. These findings underscore the importance of identifying the key ecological processes which link the changes of different traits within and between species.

## Methods

### Data compilation

We searched for peer-reviewed journal articles using ISI Web of Science and Google Scholar in March 2018 with no restriction on publication year. We focused on manipulative studies which included key global environmental changes: (1) warming, (2) eCO_2_, (3) drought, and (4) nitrogen addition. Drought here refers to rainfall reduction (field experiment) or irrigation reduction (environmentally controlled experiment). More than 4000 published articles reported changes of leaf traits (SLA, leaf nitrogen content (N), and/or net photosynthetic rate (A)) under experimental manipulations. To minimize publication bias caused by different data sources, we considered only articles with at least two of the three leaf traits in this study. After systematic screening, a total of 404 published articles were included in our database (Supplementary Table 1 and Supplementary Fig. 8). Overall, the experimental approaches included greenhouse, growth chamber, pot, garden, and field habitat. In this study, the experiments conducted in garden and field habitat were defined as field experiments. The experiments conducted in greenhouse, growth chamber, and pot were classified as environmentally controlled experiments, in which the disturbances of the other variables were minimized. As a result, this study compiled a trait plasticity database from 102 field experiments and 108 environmentally controlled experiments (Fig. 1a and Source Data). The consistency of trait relationships between field and environmentally controlled experiments has been tested before using the whole dataset in global analyses (Supplementary Note 1). The experimental condition of field experiment was characterized by MAT and mean annual precipitation, while the environmentally controlled experiment was described by experimental temperature (*T*) and relative humidity (%).

All original data were extracted from the text, tables, figures, and appendices of the publications. When data were graphically presented, GetData Graph Digitizer v2.26 was used to obtain numeric data (http://getdata-graph-digitizer.com/). Area- and mass-based measures of any traits were converted through the following relationship,

1 $$T_m = T_a \times {\rm{SLA}}$$

where *T*_*m*_ and *T*_*a*_ are the values of trait *T* expressed on per-unit mass and per-unit area, respectively; SLA is the specific leaf area (in units of cm^2^ g^−1^).

Error propagation through the conversion is calculated using the following equations:

Standard deviation (SD) of *T*_*m*_ equal to,

2 $${\rm{SD}}_{T_m} = T_m\sqrt {\left( {\frac{{{\rm{SD}}_{T_a}}}{{T_a}}} \right)^2 + \left( {\frac{{{\rm{SD}}_{\rm{{SLA}}}}}{{\rm{{SLA}}}}} \right)^2}$$

where ${\rm{SD}}_{T_m}$, ${\rm{SD}}_{T_a}$ and ${\rm{SD}}_{\rm{{SLA}}}$ are the SD of trait *T*_*m*_, *T*_*a*_, and SLA, respectively.

### Data modifications

Considering the statistical assumption of independence among observations, we used only one measurement of each species from the same study. For all the variables, if more than one observation were reported during the same experiment for the same species, a weighted average value was calculated by,

3 $$T = \mathop {\sum }\limits_{i = 1}^j \frac{{T_i}}{j}$$

with standard deviation

4 $${\rm{SD}} = \sqrt {\frac{{\mathop {\sum }\nolimits_{i = 1}^j {\rm{SD}}_i^2\left( {n_i - 1} \right)n_i}}{{\left( {\mathop {\sum }\nolimits_{i = 1}^j n_i - 1} \right)\mathop {\sum }\nolimits_{i = 1}^j n_i}}}$$

where *j* is the number of observations, *T*_*i*_, SD_*i*_, and *n*_*i*_ are the mean, SD, and sample size of the ith sampling data, respectively^65^.

For the potential nonindependence of the same species from different studies, we created another version of the dataset purged of intraspecific variation by replacing multiple observations per species with the mean of those values^3^. However, the two datasets showed same response patterns of trait relationships to different environmental changes (Supplementary Fig. 9 and Supplementary Table 11). Therefore, considering huge experimental difference (field or environmentally controlled, different treatment strength, different genotypes, etc.) of the same species from different studies, we presented the results with the larger sampling size (i.e., keeping the same species from different studies) in the main text. We refer to any observation as a species observation in the subsequent analysis.

### Statistical analysis

The effects of the global environmental changes on plant functional traits were quantified following the methods described by Hedges et al.^66^ using Metawin 2.0. The natural logarithm-transformed response ratio (RR) was used to evaluate the environmental changes effects on leaf traits:

5 $${\mathrm{{ln}RR}} = {\mathrm{ln}}\left( {\frac{{T_t}}{{T_c}}} \right)$$

where *T*_*t*_ and *T*_*c*_ are the experimental treatment mean and control mean, respectively.

The variance (ν) is estimated by:

6 $$v = \frac{{{\rm{SD}}_t^2}}{{n_tT_t^2}} + \frac{{{\rm{SD}}_c^2}}{{n_cT_c^2}}$$

where *n*_*t*_ and *n*_*c*_ represent the sample size, and SD_*t*_ and SD_*c*_ are the SD for the treatment and control variables, respectively. The weight of each RR is the reciprocal of its variance $\left( {w = \frac{1}{V}} \right)$. Then the weighted response ratio (RR_++_) is calculated as below (*m* is the number of groups and *n* is the number of comparison):

7 $${\rm{RR}}_{ + + } = \frac{{\mathop {\sum }\nolimits_{i = 1}^m \mathop {\sum }\nolimits_{j = 1}^n W_{ij}{\rm{RR}}_{ij}}}{{\mathop {\sum }\nolimits_{i = 1}^m \mathop {\sum }\nolimits_{j = 1}^n W_{ij}}}$$

with the standard error (SE) is calculated as

8 $${\mathrm{S}}\left( {\rm{{RR}}_{ + + }} \right) = \sqrt {\frac{1}{{\mathop {\sum }\nolimits_{i = 1}^m \mathop {\sum }\nolimits_{j = 1}^n W_{ij}}}}$$

Then the 95% confidence interval (95% CI) is ${\rm{RR}}_{ + + } \pm 1.96\,{\rm{S}}\left( {{\rm{RR}}_{ + + }} \right)$. The percentage changes of a variable were calculated as:

9 $${\mathrm{A}} = \left[ {\exp \left( {{\rm{RR}}_{ + + }} \right) - 1} \right] \times 100\%$$

The effects of environmental changes were evaluated as significant, if the 95% CI does not overlap zero. We also used *Q*-statistic^67^ to test the heterogeneity of the effect sizes between different functional groups (angiosperm woody vs. gymnosperm woody, dicotyledons vs. monocotyledons, and C_3_ herb vs. C_4_ herb) and growth environment (field vs environmentally controlled conditions, low strength vs high strength, and treatment duration). If *Q*_*b*_ is larger than a critical value, there would be significant difference between the categories. Statistical significance was tested at the *P* < 0.05 level.

### SMA regression

Considering the concurrent errors in both axes, we used SMA regressions to quantify allometric relationships of pairwise traits under control and treatment conditions. The DOS-based SMATR package used for SMA regressions allows testing both for homogeneity among SMA slopes via a permutation test and for differences in SMA elevation via the SMA analog of standard ANCOVA^68^. When homogeneity was demonstrated (*P* > 0.05), a common slope was estimated. Elevation homogeneity comparisons were performed only when slopes were homogeneous. Where noted in the results, log_10_ transformations were carried out on the original data and SMA regression then fitted between leaf functional traits.

### Covariance error ellipse

Covariance error ellipses were created to summarize the distribution characteristics of leaf traits along the common axis: central tendency, dispersion, and directional trends. These measures define the axes of an ellipse encompassing the distribution of features. The covariance error ellipse was drawn with Matlab^69^.

### Reporting summary

Further information on research design is available in the Nature Research Reporting Summary linked to this article.

## Supplementary information

Supplementary Information

Reporting Summary

## Data Availability

The authors declare that the data supporting the findings of this study are available within the paper and its supplementary information files. Source Data are provided with this paper.

## Source data

Source Data

## Code availability

No custom code or mathematical algorithms were developed for this study. Only existing packages and software were used for the analysis, which can be found in the “Methods.”

## References

[1] Reich PB, Walters MB, Ellsworth DS (1997). From tropics to tundra: global convergence in plant functioning. Proc. Natl Acad. Sci. USA.

[2] Wright IJ (2004). The worldwide leaf economics spectrum. Nature.

[3] Osnas JL, Lichstein JW, Reich PB, Pacala SW (2013). Global leaf trait relationships: mass, area, and the leaf economics spectrum. Science.

[4] Wright IJ (2005). Modulation of leaf economic traits and trait relationships by climate. Glob. Ecol. Biogeogr..

[5] Reich PB (2014). The world-wide ‘fast-slow’ plant economics spectrum: a traits manifesto. J. Ecol..

[6] Reich PB, Flores-Moreno H (2017). Peeking beneath the hood of the leaf economics spectrum. New Phytol..

[7] Reich PB (2012). Key canopy traits drive forest productivity. Proc. R. Soc. B.

[8] Sakschewski B (2015). Leaf and stem economics spectra drive diversity of functional plant traits in a dynamic global vegetation model. Glob. Chang Biol..

[9] van Bodegom PM, Douma JC, Verheijen LM (2014). A fully traits-based approach to modeling global vegetation distribution. Proc. Natl Acad. Sci. USA.

[10] Butler EE (2017). Mapping local and global variability in plant trait distributions. Proc. Natl Acad. Sci. USA.

[11] Sakschewski B (2016). Resilience of Amazon forests emerges from plant trait diversity. Nat. Clim. Change.

[12] Wang H (2017). Towards a universal model for carbon dioxide uptake by plants. Nat. Plants.

[13] Wang YP (2012). Correlations among leaf traits provide a significant constraint on the estimate of global gross primary production. Geophys. Res. Lett..

[14] Reichstein M, Bahn M, Mahecha MD, Kattge J, Baldocchi DD (2014). Linking plant and ecosystem functional biogeography. Proc. Natl Acad. Sci. USA.

[15] Anderegg L (2018). Within-species patterns challenge our understanding of the leaf economics spectrum. Ecol. Lett..

[16] Rozendaal DMA, Hurtado VH, Poorter L (2006). Plasticity in leaf traits of 38 tropical tree species in response to light; relationships with light demand and adult stature. Funct. Ecol..

[17] Doughty CE (2018). Tropical forest leaves may darken in response to climate change. Nat. Ecol. Evol..

[18] Yang Y (2019). Quantifying leaf-trait covariation and its controls across climates and biomes. New Phytol..

[19] Reich PB, Wright IJ, Lusk CH (2007). Predicting leaf physiology from simple plant and climate attributes: a global GLOPNET analysis. Ecol. Appl..

[20] Meng TT (2015). Responses of leaf traits to climatic gradients: adaptive variation versus compositional shifts. Biogeosciences.

[21] Fyllas NM (2014). Analysing Amazonian forest productivity using a new individual and trait-based model (TFS v. 1). Geosci. Model Dev..

[22] Weng E, Farrior CE, Dybzinski R, Pacala SW (2017). Predicting vegetation type through physiological and environmental interactions with leaf traits: evergreen and deciduous forests in an earth system modeling framework. Glob. Change Biol..

[23] Lusk CH, Reich PB, Montgomery RA, Ackerly DD, Cavender-Bares J (2008). Why are evergreen leaves so contrary about shade?. Trends Ecol. Evol..

[24] Fisher RA (2015). Taking off the training wheels: the properties of a dynamic vegetation model without climate envelopes, CLM4.5 (ED). Geosci. Model Dev..

[25] Verheijen LM (2015). Inclusion of ecologically based trait variation in plant functional types reduces the projected land carbon sink in an earth system model. Glob. Change Biol..

[26] Yang Y, Zhu Q, Peng C, Wang H, Chen H (2015). From plant functional types to plant functional traits: a new paradigm in modelling global vegetation dynamics. Prog. Phys. Geog..

[27] Williams JW, Jackson ST, Kutzbach JE (2007). Projected distributions of novel and disappearing climates by 2100 AD. Proc. Natl Acad. Sci. USA.

[28] van Bodegom PM (2012). Going beyond limitations of plant functional types when predicting global ecosystem–atmosphere fluxes: exploring the merits of traits-based approaches. Glob. Ecol. Biogeogr..

[29] Keenan TF, Niinemets Ülo (2016). Global leaf trait estimates biased due to plasticity in the shade. Nat. Plants.

[30] Kovenock M, Swann AL (2018). Leaf trait acclimation amplifies simulated climate warming in response to elevated carbon dioxide. Glob. Biogeochem. Cycles..

[31] Poorter H, Niinemets Ü, Poorter L, Wright IJ, Villar R (2009). Causes and consequences of variation in leaf mass per area (LMA): a meta-analysis. New Phytol..

[32] De Frenne P (2015). Light accelerates plant responses to warming. Nat. Plants.

[33] Marquet PA (2005). Scaling and power-laws in ecological systems. J. Exp. Biol..

[34] Wright IJ, Reich PB, Westoby M (2001). Strategy shifts in leaf physiology, structure and nutrient content between species of high- and low-rainfall and high- and low-nutrient habitats. Funct. Ecol..

[35] Reich PB (2018). Effects of climate warming on photosynthesis in boreal tree species depend on soil moisture. Nature.

[36] Lloyd J, Bloomfield K, Domingues TF, Farquhar GD (2013). Photosynthetically relevant foliar traits correlating better on a mass vs an area basis: of ecophysiological relevance or just a case of mathematical imperatives and statistical quicksand?. New Phytol..

[37] Westoby M, Reich PB, Wright IJ (2013). Understanding ecological variation across species: area-based vs mass-based expression of leaf traits. New Phytol..

[38] Díaz S (2016). The global spectrum of plant form and function. Nature.

[39] Poorter H (2016). Pampered inside, pestered outside? Differences and similarities between plants growing in controlled conditions and in the field. New Phytol..

[40] Huang M (2019). Air temperature optima of vegetation productivity across global biomes. Nat. Ecol. Evol..

[41] Rustad L (2001). A meta-analysis of the response of soil respiration, net nitrogen mineralization, and aboveground plant growth to experimental ecosystem warming. Oecologia.

[42] Wu Z, Dijkstra P, Koch GW, Peñuelas J, Hungate BA (2011). Responses of terrestrial ecosystems to temperature and precipitation change: a meta-analysis of experimental manipulation. Glob. Change Biol..

[43] León-Sánchez L (2020). Altered leaf elemental composition with climate change is linked to reductions in photosynthesis, growth and survival in a semi-arid shrubland. J. Ecol..

[44] Temme AA (2017). Increases in CO_2_ from past low to future high levels result in “slower” strategies on the leaf economic spectrum. Perspect. Plant Ecol..

[45] Salguero-Gómez R (2017). Applications of the fast-slow continuum and reproductive strategy framework of plant life histories. New Phytol..

[46] Ainsworth EA, Long SP (2005). What have we learned from 15 years of free-air CO_2_ enrichment (FACE)? A meta-analytic review of the responses of photosynthesis, canopy properties and plant production to rising CO_2_. New Phytol..

[47] Luo Y (2004). Progressive nitrogen limitation of ecosystem responses to rising atmospheric carbon dioxide. Bioscience.

[48] Dwyer JM, Hobbs RJ, Mayfield MM (2014). Specific leaf area responses to environmental gradients through space and time. Ecology.

[49] Firn J (2019). Leaf nutrients, not specific leaf area, are consistent indicators of elevated nutrient inputs. Nat. Ecol. Evol..

[50] Wang C, McCormack ML, Guo D, Li J (2019). Global meta-analysis reveals different patterns of root tip adjustments by angiosperm and gymnosperm trees in response to environmental gradients. J. biogeogr..

[51] Taylor SH (2010). Ecophysiological traits in C_3_ and C_4_ grasses: a phylogenetically controlled screening experiment. New Phytol..

[52] Galmes J, Kapralov MV, Copolovici LO, Hermida-Carrera C, Niinemets Ü (2015). Temperature responses of the Rubisco maximum carboxylase activity across domains of life: phylogenetic signals, trade-offs, and importance for carbon gain. Photosynth. Res..

[53] Shao J (2019). Plant evolutionary history mainly explains the variance in biomass responses to climate warming at a global scale. New Phytol..

[54] Osnas JL (2018). Divergent drivers of leaf trait variation within species, among species, and among functional groups. Proc. Natl Acad. Sci. USA.

[55] Onoda Y (2017). Physiological and structural tradeoffs underlying the leaf economics spectrum. New Phytol..

[56] Feng YL (2009). Evolutionary tradeoffs for nitrogen allocation to photosynthesis versus cell walls in an invasive plant. Proc. Natl Acad. Sci. USA.

[57] Onoda Y (2011). Global patterns of leaf mechanical properties. Ecol. Lett..

[58] Leakey AD (2009). Elevated CO_2_ effects on plant carbon, nitrogen, and water relations: six important lessons from FACE. J. Exp. Bot..

[59] Bauer GA, Berntson GM, Bazzaz FA (2001). Regenerating temperate forests under elevated CO_2_ and nitrogen deposition: comparing biochemical and stomatal limitation of photosynthesis. New Phytol..

[60] Martin KC (2010). Nitrogen fertilization enhances water-use efficiency in a saline environment. Plant Cell Environ..

[61] Oleson, K. W. et al. *Technical Description of Version 4.5 of the Community Land Model (CLM)*. Ncar Technical Note NCAR/TN-503+STR, National Center for Atmospheric Research, Boulder, CO, 422 pp (2013). 10.5065/D6RR1W7M.

[62] Cui E (2019). Vegetation functional properties determine uncertainty of simulated ecosystem productivity: a traceability analysis in the East Asian monsoon region. Glob. Biogeochem. Cycles.

[63] Fry EL (2019). Using plant, microbe, and soil fauna traits to improve the predictive power of biogeochemical models. Methods Ecol. Evol..

[64] He N (2019). Ecosystem traits linking functional traits to macroecology. Trends Ecol. Evol..

[65] Liang J, Qi X, Souza L, Luo Y (2016). Processes regulating progressive nitrogen limitation under elevated carbon dioxide: a meta-analysis. Biogeosciences.

[66] Hedges LV, Gurevitch J, Curtis PS (1999). The meta-analysis of response ratios in experimental ecology. Ecology.

[67] Gurevitch. J. & Hedges, L. V. Meta-analysis: combining the results of independent experiments. In: Scheiner, S. M. & Gurevitch, J. (eds) *Design and Analysis of Ecological Experiments*. (Chapman and Hall, New York, 1993). pp 378–425.

[68] Wright IJ (2005). Assessing the generality of global leaf trait relationships. New Phytol..

[69] Spruyt, V. How to Draw an Error Ellipse Representing the Covariance Matrix? *Computer Vision for Dummies*, 2014. https://www.visiondummy.com/2014/04/draw-error-ellipse-representing-covariance-matrix/.

