# Supplementary material for: Robust leaf trait relationships across species under global environmental changes
Source: Nat Commun. 2020 Jun 12;11:2999. doi: 10.1038/s41467-020-16839-9 (PMC7293315; doi:10.1038/s41467-020-16839-9)
Supplement: Supplementary file 1 — Supplementary Information [file 41467_2020_16839_MOESM1_ESM.pdf]

Supplementary Information

Robust leaf trait relationships across species under global environmental changes

Cui *et al*.

Contents

**Supplementary**	**Figure 1-9**
**Supplementary**	**Table 1-11**
**Supplementary**	**Note 1-2**

Supplementary Figures

Supplementary Figure 1: The environmental conditions for the collected experiments.

Supplementary Figure 2: Changes in percentage of leaf traits under global environmental changes for field and environmentally controlled groups.

Supplementary Figure 3: Dependence of leaf traits' response to global environmental factors on treatment strengths and duration.

Supplementary Figure 4: Response of area- and mass-based leaf trait relationships under global environmental changes.

Supplementary Figure 5: Comparison of trait relationships between functional groups.

Supplementary Figure 6: Comparison of changes in elevations of leaf trait relationships between functional groups.

Supplementary Figure 7: Comparison of changes in elevations of leaf trait relationships between field and controlled experiments as well as low and high treatment strength.

Supplementary Figure 8: The PRISMA flow diagram for the selection of published papers.

Supplementary Figure 9: Response of leaf trait relationships to global environmental changes with and without species overlap.

Supplementary Tables

Supplementary Table 1: Number of species and range of trait values under each global change driver included in the database.

Supplementary Table 2: Heterogeneity of effect sizes under different treatment strength and duration.

Supplementary Table 3: Analysis of SMA regression values for mass-based log-transformed trait relationships under control and treatment conditions.

Supplementary Table 4: Analysis of SMA regression values for area-based log-transformed trait relationships under control and treatment conditions.

Supplementary Table 5: Comparison of SMA regression values for mass-based trait relationships (of the form $\log y = k * \log x + b$) between functional groups.

Supplementary Table 6: Analysis of SMA regression values for log-transformed trait relationships under control and treatment conditions between angiosperm woody and gymnosperm woody.

Supplementary Table 7: Analysis of SMA regression values for log-transformed trait relationships under control and treatment conditions between dicotyledons and monocotyledons.

Supplementary Table 8: Analysis of SMA regression values for log-transformed trait relationships under control and treatment between C_3 herb and C_4 herb.

Supplementary Table 9: Analysis of SMA regression values for log-transformed trait

relationships under control and treatment conditions between field and controlled experiments.

Supplementary Table 10: Analysis of SMA regression values for log-transformed trait relationships under control and treatment conditions between low strength and high strength.

Supplementary Table 11: Analysis of SMA regression values for log-transformed trait relationships under control and treatment conditions between with and without species overlap.

Supplementary Notes

Supplementary Note 1: The consistency of trait relationships between field and environmentally controlled experiments.

Supplementary Note 2: The presentation of species-level response into trait-trait space.

Supplementary Figures

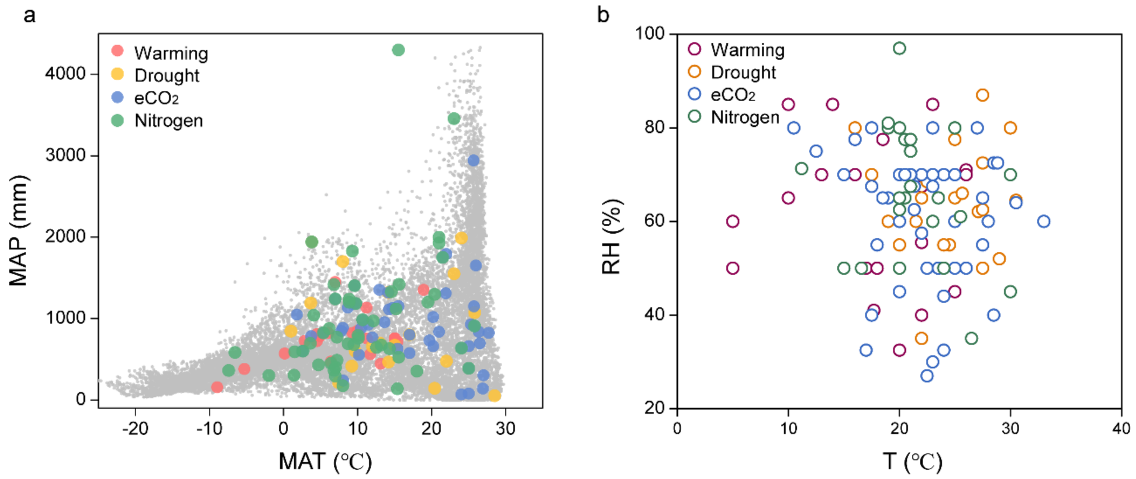

Supplementary Figure 1. The environmental conditions for the collected experiments. Note that the field experiments are characterized by mean annual temperature (MAT) and mean annual precipitation (MAP) (a), while the environmentally controlled experiment are characterized by experimental temperature (T) and relative humidity (%) (b). The grey points represent the global land MAT and MAP.

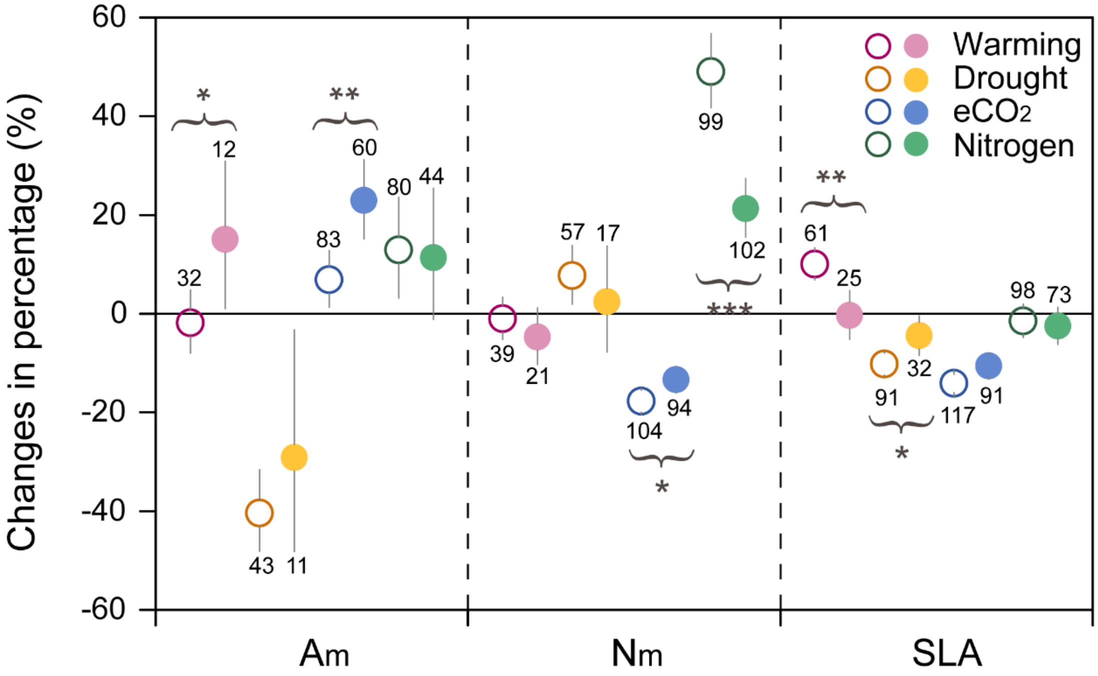

Supplementary Figure 2. Changes in percentage of leaf traits under global environmental changes for field and environmentally controlled groups. The filled circles represent the field experiments and the open circles for the environmentally controlled experiments. Circles represent the global mean changes, and error bars are 95% credible intervals on the mean. The number of observations for each category is shown near the bar. Between-group heterogeneity of effect size under different environmental changes is tested by Q-statistic and shown: $*P < 0.05$; $**P < 0.01$; $***P < 0.001$.

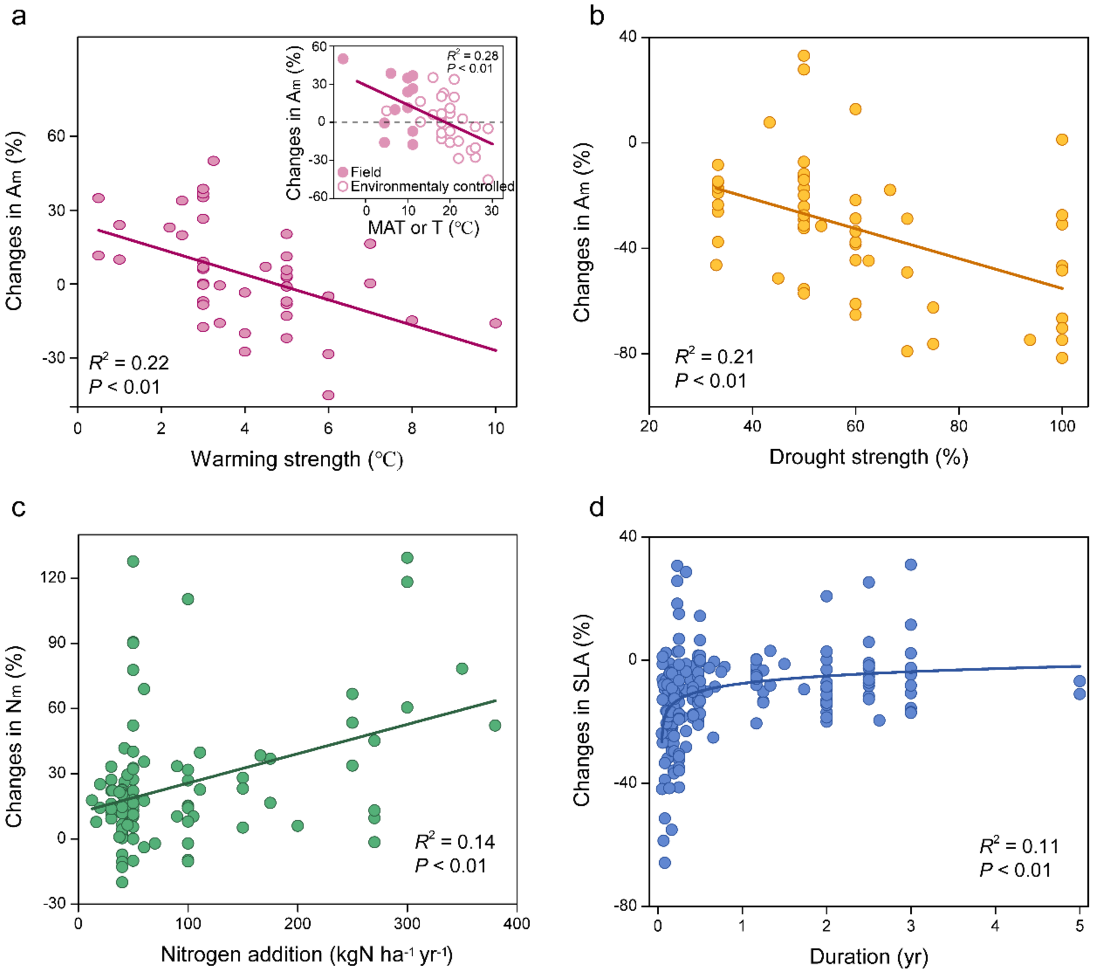

Supplementary Figure 3. Dependence of leaf traits' response to global environmental factors on treatment strengths (a-c) and duration (d). Inset in Figure S3a, dependence of changes in A_m on temperature conditions of plants. The filled circles represent the observations of field experiments and the open circles for the observations of environmentally controlled experiments. The statistics information is shown in Supplementary Table 2. Note that drought strength here is defined as the reduction in percentage (%) of water availability (precipitation, irrigation, soil moisture, field capacity, etc.).

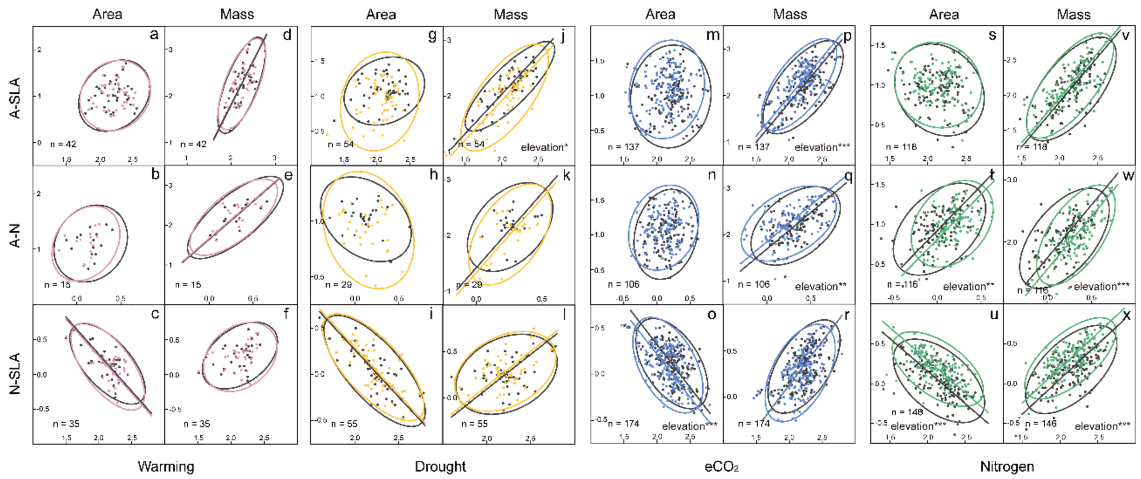

Supplementary Figure 4. Response of area- and mass-based leaf trait relationships under global environmental changes. The black and colored ellipses indicate distribution of control and treatment observations, respectively. Ellipses are 95% confidence level of the original scatters. The bold lines represent SMA regressions of leaf trait relationships. The number of observations is shown near ellipses. The homogeneity among SMA slopes via a permutation test and for differences in SMA elevation via the SMA analogue of standard ANCOVA. The statistics information is shown in Supplementary Table 4. Note that the relationship changes of trait combinations that without significant correlations are not detected. Significance of changes in elevations: $*P < 0.05$; $**P < 0.01$; $***P < 0.001$.

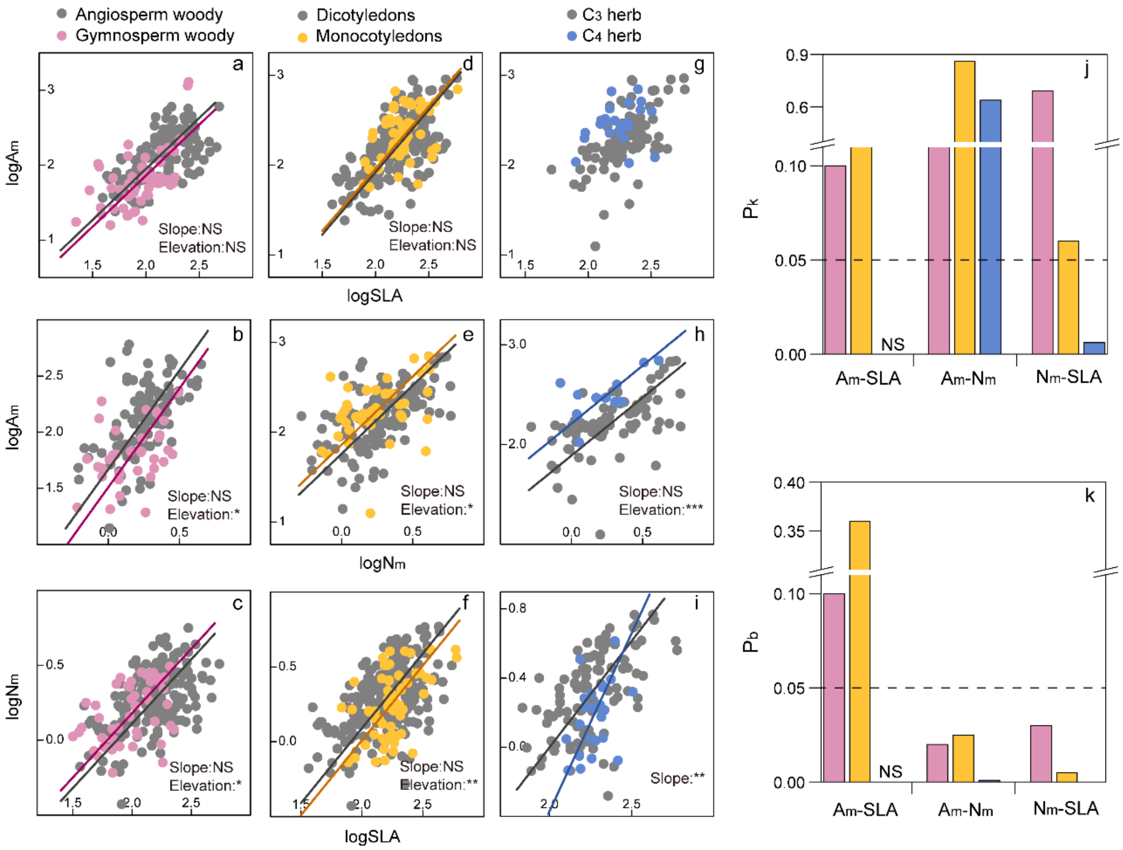

Supplementary Figure 5. (a-i) Comparison of trait relationships between functional groups. The homogeneity among SMA slopes via a permutation test and for differences in SMA elevation via the SMA analogue of standard ANCOVA. The statistics information is shown in Supplementary Table 5. The Significance: NS: $P > 0.05$; $*P < 0.05$; $**P < 0.01$; $***P < 0.001$. (j, k) The statistical significance of functional difference in slope (P_k) or elevation (P_b) of trait relationships. The number of observations is shown in the panel.

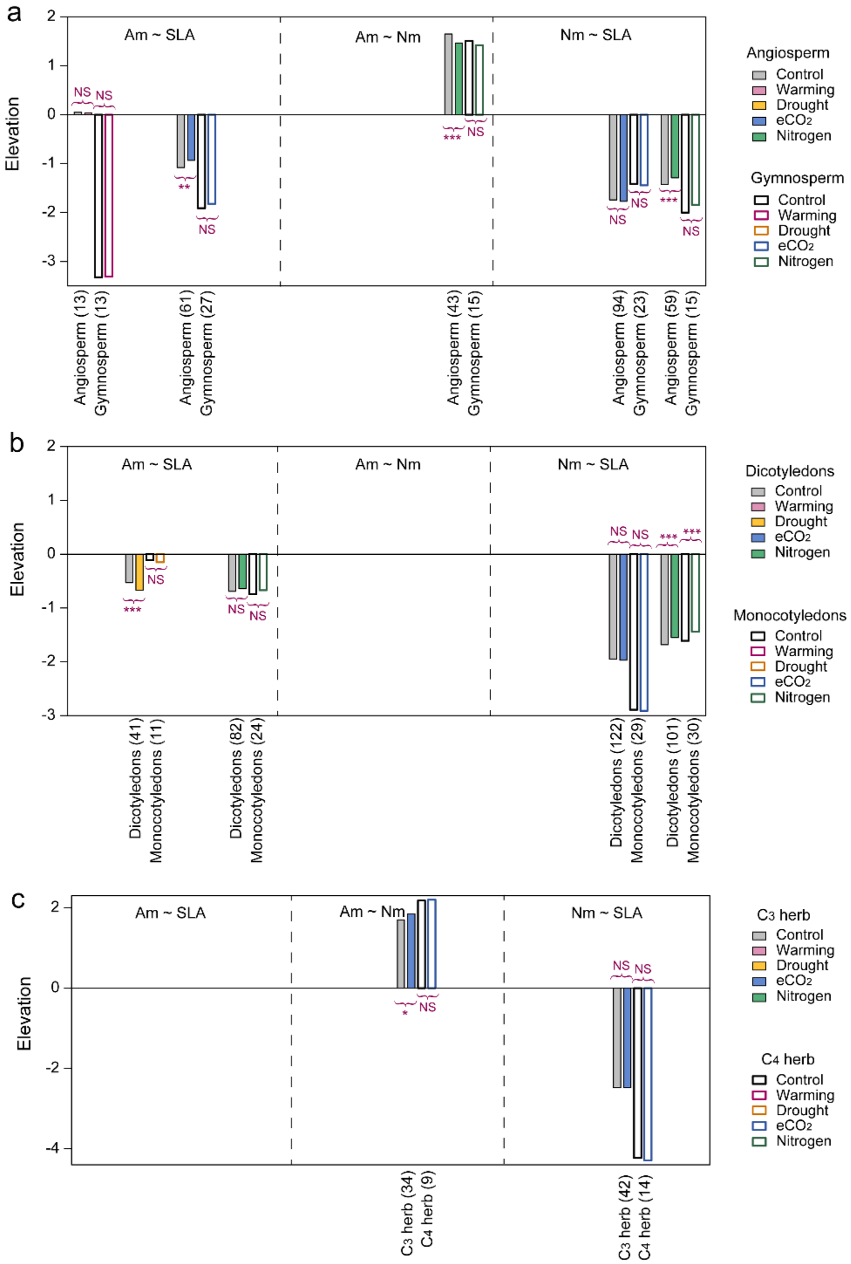

Supplementary Figure 6. Comparison of changes in elevations of leaf trait relationships between angiosperm woody and gymnosperm woody (a), dicotyledons and monocotyledons (b) as well as C_3 herb and C_4 herb (c). The homogeneity among SMA slopes via a permutation test and for differences in SMA elevation via the SMA analogue of standard ANCOVA. The statistics information is shown in Supplementary Table 6-8. The number of observations for each category is shown near the category. Note that the relationship changes of trait combinations that without significant correlations are not detected. Significance: NS: $P > 0.05$; *$P < 0.05$; **$P < 0.01$; ***$P < 0.001$.

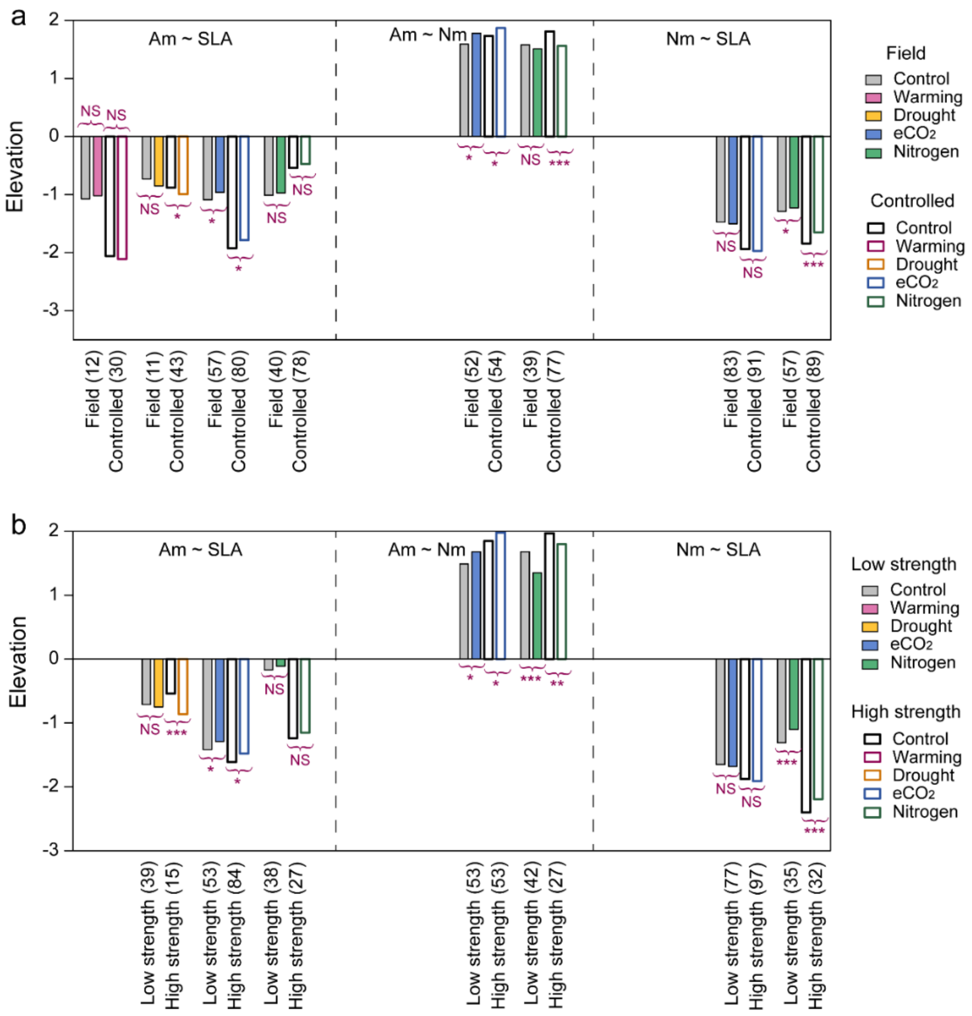

Supplementary Figure 7. Comparison of changes in elevations of leaf trait relationships between field and controlled experiments (a) as well as low and high treatment strength (b). The homogeneity among SMA slopes via a permutation test and for differences in SMA elevation via the SMA analogue of standard ANCOVA. The statistics information is shown in Supplementary Table 9 and 10. The partition criterion for the treatment strength is as follows: Warming (low: <= 3 °C; high > 3°C); Drought (low < 70%; high >= 70%); eCO_2 (low < 1.8 fold; high >=1.8 fold); Nitrogen (low: < 5 mmol/l; high >= 5 mmol/l). The number of observations is shown near the category. Note that the relationship changes of trait combinations that without significant correlations are not detected. Significance: NS: $P > 0.05$; *$P < 0.05$; **$P < 0.01$; ***$P < 0.001$.

Identification

Literature search: *Web of Science* and *Google Scholar*

Meeting titles:

(1) (nitrogen deposition or nitrogen addition or nitrogen application or nitrogen or nutrient fertilization or nitrogen supply or nitrogen enrichment or nitrogen availability) and (photosynthetic or photosynthesis or response or leaf or specific leaf area or leaf nitrogen or productivity or growth or production or physiological or leaf trait or physiology)

(2) (elevated CO_2 or CO_2 or CO_2 concentration or CO_2 enrichment or CO_2 availability) and (photosynthetic or photosynthesis or response or leaf or leaf nitrogen or productivity or growth or production or physiological or leaf trait or physiology)

(3) (warming or temperature) and (photosynthetic or photosynthesis or response or leaf or leaf nitrogen or productivity or growth or production or physiological or leaf trait or physiology)

(4) (drought or precipitation or water or arid or dry) and (photosynthetic or photosynthesis or response or leaf or leaf nitrogen or productivity or growth or production or physiological or leaf trait or physiology)

Limits: English-language articles, before March 2018

Search results: (n =52734)

↓

Screening

Records screened on Research Domains, Document types and Source titles (n=9260)

Excluded (n = 44474)

Not Science Technology: 6598

Not Journal Articles: 8946

Not categories related to ecology, geophysics, plant, soil and environment: 29920

↓

Eligibility

Full-text articles review and assessed on criteria (n=4168)

Excluded: (n=5092)

Not species level: 397

Not target traits: 4678

Duplicated results in various studies: 17

↓

Species criteria (n = 404)

Excluded: (n=3764)

Not seed plant: 28

Incomplete Statistics or Experimental information: 290

At least two of the target traits: 3446

↓

Included

Studies included in synthesis: Nitrogen (n=99); eCO_2 (n=134); Warming (n=82); Drought (n=89)

Supplementary Figure 8. The PRISMA flow diagram for the selection of published papers.

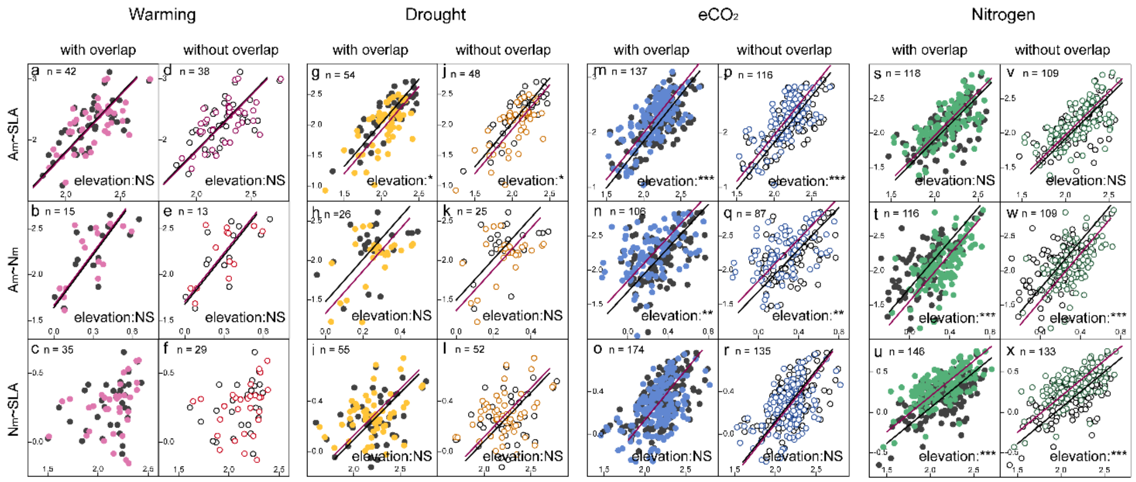

Supplementary Figure 9. Response of leaf trait relationships to global environmental changes with and without species overlap. The black circles and the colored circles represent the observations from control and treatment experiments, respectively. The bold lines represent SMA regressions of leaf trait relationships. The homogeneity among SMA slopes via a permutation test and for differences in SMA elevation via the SMA analogue of standard ANCOVA. Note that the relationship changes of trait combinations that without significant correlations are not detected. Significance: NS: $P > 0.05$; $*P < 0.05$; $**P < 0.01$; $***P < 0.001$.

Supplementary Tables

Supplementary Table 1. Number of species and range of trait values under each global change driver included in the database. The compiled articles in this global trait database list in Source Data.

Global change driver	Leaf traits	mean value	sd	min	max	species
Warming	SLA (cm^2 g^{-1})	175.08	82.91	31.75	580.70	86
	N_m (%)	2.21	1.09	0.68	4.60	60
	A_m (nmol g^{-1} s^{-1})	246.72	251.44	18.14	1295.28	44
eCO_2	SLA (cm^2 g^{-1})	186.82	95.70	31.75	588.24	208
	N_m (%)	2.42	1.14	0.69	6.09	198
	A_m (nmol g^{-1} s^{-1})	221.93	196.15	12.50	1152.54	143
Drought	SLA (cm^2 g^{-1})	217.69	171.55	22.15	1241.62	123
	N_m (%)	2.13	0.96	0.68	4.73	74
	A_m (nmol g^{-1} s^{-1})	186.63	111.68	17.38	511.12	54
Nitrogen	SLA (cm^2 g^{-1})	161.85	81.16	25.77	422.23	171
	N_m (%)	1.71	0.81	0.22	5.15	201
	A_m (nmol g^{-1} s^{-1})	149.34	108.11	13.98	678.80	124

Supplementary Table 2. Heterogeneity of effect sizes under different treatment strength and duration. Between-group heterogeneity of effect size under different environmental changes is tested by *Q*-statistic

Treatment	Variable	A_m			N_m			SLA		
		Slope	Q_b	*P*	Slope	Q_b	*P*	Slope	Q_b	*P*
Warming	Treatment strength	-0.051	16.6	**<0.001**	0.006	1.1	0.29	0.019	8.6	0.06
	Duration	0.035	0.3	0.61	-0.028	1.7	0.19	-0.028	3.8	0.05
Drought	Treatment strength	-0.011	15.8	**<0.001**	0.001	2.3	0.13	0.001	3.8	0.05
	Duration	0.070	0.2	0.69	-0.022	2.0	0.16	0.014	0.2	0.66
eCO_2	Treatment strength	-0.066	0.5	0.48	-0.059	1.4	0.23	-0.075	3.7	0.05
	Duration	0.002	0	0.94	0.029	7.9	0.05	0.041	21	**<0.001**
Nitrogen	Treatment strength (mmol L^{-1})	-0.072	1.7	0.19	-0.020	0.3	0.57	-0.011	1.1	0.30
	Treatment strength (kgN $ha^{-1}yr^{-1}$)	0.001	1.6	0.21	0.001	20.8	**<0.001**	0.0002	0.7	0.42
	Duration	-0.020	0.3	0.57	-0.014	3.7	0.05	0.0001	0.0	0.98

Significance: $*P < 0.05$; $**P < 0.01$; $***P < 0.001$. The results of nitrogen addition were divided into two groups based on the unit of applied dose.

Supplementary Table 3. Analysis of SMA regression values for mass-based trait relationships (of the form $\log y = k * \log x + b$) under control and treatment conditions (warming, eCO_2, drought and nitrogen). The homogeneity among SMA slopes via a permutation test and for differences in SMA elevation via the SMA analogue of standard ANCOVA.

Correlations	Treatment	r^2	P	Group slope (k) 95% CI	Common slope (k) 95% CI	Elevation (b) 95% CI	Test of common lines	
							P_k	P_b
$A_m \sim N_m$	control	0.55	**<0.01**	1.90 (1.28, 2.82)	1.91 (1.44, 2.54)	1.64 (1.42, 1.86)	0.95	0.80
	warming	0.46	**<0.01**	1.93 (1.26, 2.96)		1.66 (1.45, 1.88)		
$A_m \sim SLA$	control	0.48	**<0.001**	1.92 (1.53, 2.41)	1.88 (1.59, 2.22)	-1.95 (-2.65, -1.24)	0.80	0.73
	warming	0.42	**<0.001**	1.84 (1.44, 2.34)		-1.97 (-2.68, -1.25)		
$N_m \sim SLA$	control	0.16	**0.02**					
	warming	0.08	0.10					
$A_m \sim N_m$	control	0.21	**<0.001**	1.67 (1.40, 1.98)	1.58 (1.40, 1.79)	1.69 (1.60, 1.78)	0.39	**<0.01**
	eCO_2	0.20	**<0.001**	1.50 (1.26, 1.78)		1.84 (1.76, 1.93)		
$A_m \sim SLA$	control	0.43	**<0.001**	1.65 (1.45, 1.88)	1.70 (1.55, 1.86)	-1.56 (-1.90, -1.21)	0.57	**<0.001**
	eCO_2	0.45	**<0.001**	1.74 (1.54, 1.98)		-1.42 (-1.76, -1.09)		
$N_m \sim SLA$	control	0.32	**<0.001**	0.92 (0.82, 1.05)	0.94 (0.85, 1.03)	-1.75 (-1.95, -1.55)	0.69	0.96
	eCO_2	0.31	**<0.001**	0.96 (0.83, 1.11)		-1.75 (-1.94, -1.55)		
$A_m \sim N_m$	control	0.17	**0.04**	2.47 (1.70, 3.59)	2.68 (2.07, 3.47)	1.48 (1.25, 1.70)	0.54	0.11
	drought	0.25	**<0.01**	2.89 (2.02, 4.13)		1.33 (1.10, 1.55)		

A_m ~ SLA	control	0.68	**<0.001**	1.32 (1.12, 1.54)	1.42 (1.26, 1.62)	-0.81 (-1.19, -0.43)	0.12	**0.02**
	drought	0.48	**<0.001**	1.61 (1.32, 1.96)		-0.93 (-1.30, -0.56)		
N_m ~ SLA	control	0.09	**0.02**	0.75 (0.58, 0.97)	0.75 (0.63, 0.90)	-1.40 (-1.72, -1.09)	0.99	0.39
	drought	0.13	**<0.01**	0.75 (0.58, 0.97)		-1.37 (-1.68, -1.06)		
A_m ~ N_m	control	0.32	**<0.001**	1.60 (1.37, 1.86)	1.71 (1.53, 1.90)	1.75 (1.68, 1.81)	0.23	**<0.001**
	nitrogen	0.36	**<0.001**	1.82 (1.57, 2.10)		1.56 (1.49, 1.64)		
A_m ~ SLA	control	0.42	**<0.001**	1.26 (1.09, 1.44)	1.28 (1.17, 1.41)	-0.66 (-0.93, -0.40)	0.68	0.06
	nitrogen	0.50	**<0.001**	1.31 (1.15, 1.49)		-0.60 (-0.87, -0.34)		
N_m ~ SLA	control	0.34	**<0.001**	0.92 (0.80, 1.05)	0.88 (0.80, 0.96)	-1.70 (-1.87, -1.53)	0.38	**<0.001**
	nitrogen	0.43	**<0.001**	0.85 (0.75, 0.96)		-1.56 (-1.73, -1.39)		

Values represent the coefficient of determination (r^2), significance (P), slope (k) and elevation (b) of the trait relationships, and the statistical significance of the effect of global environmental changes on the slope (P_k) or elevation (P_b) of trait relationships.

Supplementary Table 4. Analysis of SMA regression values for area-based trait relationships (of the form $\log y = k * \log x + b$) under control and treatment conditions (warming, eCO_2, drought and nitrogen). The homogeneity among SMA slopes via a permutation test and for differences in SMA elevation via the SMA analogue of standard ANCOVA.

Correlations	Treatment	r^2	P	Group slope (k) 95% CI	Common slope (k) 95% CI	Elevation (b) 95% CI	Test of common lines P_k	P_b
$A_a \sim N_a$	control	0.03	0.55					
	warming	0.07	0.35					
$A_a \sim SLA$	control	0.05	0.14					
	warming	0.02	0.40					
$N_a \sim SLA$	control	0.41	**<0.001**	-1.01 (-1.33, -0.78)	-1.07 (-1.29, -0.88)	2.42 (1.99, 2.86)	0.59	0.49
	warming	0.42	**<0.001**	-1.12 (-1.47, -0.86)		2.39 (1.95, 2.83)		
$A_a \sim N_a$	control	0.03	0.05					
	eCO_2	0.04	**0.03**					
$A_a \sim SLA$	control	0.004	0.48					
	eCO_2	0.01	0.17					
$N_a \sim SLA$	control	0.26	**<0.001**	-0.89 (-1.01, -0.78)	-0.92 (-1.02, -0.84)	2.16 (1.96, 2.36)	0.42	**<0.001**
	eCO_2	0.29	**<0.001**	-0.97 (-1.12, -0.83)		2.05 (1.86, 2.25)		
$A_a \sim N_a$	control	0.10	0.09					
	drought	0.05	0.23					

$A_a \sim SLA$	control	0.05	0.12					
	drought	0.02	0.26					
$N_a \sim SLA$	control	0.53	**<0.001**	-1.04 (-1.26, -0.86)	-1.02 (-1.17, 0.90)	2.31 (2.00, 2.62)	0.79	0.42
	drought	0.52	**<0.001**	-1.00 (-1.22, -0.83)		2.34 (2.03, 2.65)		
$A_a \sim N_a$	control	0.20	**<0.001**	1.10 (0.93, 1.30)	1.14 (1.01, 1.28)	0.92 (0.87, 0.96)	0.62	**<0.01**
	nitrogen	0.11	**<0.001**	1.17 (0.98, 1.40)		0.81 (0.76, 0.6)		
$A_a \sim SLA$	control	0.03	0.05					
	nitrogen	0.01	0.36					
$N_a \sim SLA$	control	0.28	**<0.001**	-0.87 (-1.00, -0.75)	-0.82 (-0.90, -0.74)	1.80 (1.62, 1.97)	0.26	**<0.001**
	nitrogen	0.33	**<0.001**	-0.78 (-0.88, -0.68)		1.93 (1.76, 2.11)		

Values represent the coefficient of determination (r^2), significance (P), slope (k) and elevation (b) of the trait relationships, and the statistical significance of the effect of global environmental changes on the slope (P_k) or elevation (P_b) of trait relationships.

Supplementary Table 5. Comparison of SMA regression values for mass-based trait relationships (of the form $\log y = k * \log x + b$) between functional groups. The homogeneity among SMA slopes via a permutation test and for differences in SMA elevation via the SMA analogue of standard ANCOVA.

Correlations	Group	r^2	*P*	Group slope (*k*) 95% CI	Common slope (*k*) 95% CI	Elevation (*b*) 95% CI	Test of common lines	
							P_k	P_b
$A_m \sim SLA$	angiosperm	0.41	**<0.001**	1.29 (1.14,1.46)	1.36 (1.22, 1.51)	-0.77 (-1.09, -0.46)	0.10	0.10
	gymnosperm	0.33	**<0.001**	1.60 (1.28, 2.00)		-0.86 (-1.16, -0.56)		
$A_m \sim N_m$	angiosperm	0.31	**<0.001**	1.86 (1.60, 2.16)	1.76 (1.54, 2.02)	1.67 (1.59, 1.75)	0.11	**<0.05**
	gymnosperm	0.15	**0.02**	1.40 (1.02, 1.91)		1.51 (1.39, 1.63)		
$N_m \sim SLA$	angiosperm	0.31	**<0.001**	0.85 (0.76, 0.96)	0.86 (0.79, 0.96)	-1.61 (-1.81, -1.41)	0.69	**<0.05**
	gymnosperm	0.23	**<0.001**	0.91 (0.70, 1.17)		-1.54 (-1.73, -1.35)		
$A_m \sim SLA$	dicot	0.41	**<0.001**	1.36 (1.23, 1.50)	1.39 (1.27, 1.53)	-0.86 (-1.14, -0.57)	0.28	0.36
	monocot	0.16	**<0.001**	1.56 (1.24, 1.95)		-0.82 (-1.12, -0.52)		
$A_m \sim N_m$	dicot	0.35	**<0.001**	1.53 (1.36, 1.72)	1.52 (1.36, 1.70)	1.76 (1.70, 1.82)	0.86	**<0.05**
	monocot	0.12	**0.02**	1.49 (1.14, 1.95)		1.86 (1.76, 1.97)		
$N_m \sim SLA$	dicot	0.29	**<0.001**	0.96 (0.87, 1.05)	1.00 (0.91, 1.09)	-1.91 (-2.10, -1.71)	0.06	**<0.01**
	monocot	0.24	**<0.001**	1.19 (0.97, 1.46)		-1.99 (-2.20, -1.78)		
$A_m \sim SLA$	C3	0.34	**<0.001**					
	C4	0.07	0.18					

$A_m \sim N_m$	C3	0.35	**<0.001**	1.18 (0.99, 1.40)	1.16 (0.99, 1.36)	1.89 (1.81,1.97)	0.64	**<0.001**
	C4	0.38	**<0.05**	1.05 (0.68, 1.63)		2.21 (2.10, 2.32)		
$N_m \sim SLA$	C3	0.32	**<0.001**	1.23 (1.06, 1.43)		-2.46 (-2.89, -2.04)	**<0.01**	
	C4	0.15	**<0.05**	2.06 (1.45, 2.92)		-4.49 (-6.16, -2.82)		

Values represent the coefficient of determination (r^2), significance (P), slope (k) and elevation (b) of the trait relationships, and the statistical significance of the effect of global environmental changes on the slope (P_k) or elevation (P_b) of trait relationships.

Supplementary Table 6. Analysis of SMA regression values for trait relationships (of the form $\log y = k * \log x + b$) under control and treatment conditions (warming, eCO_2, drought and nitrogen) between angiosperm woody and gymnosperm woody. The homogeneity among SMA slopes via a permutation test and for differences in SMA elevation via the SMA analogue of standard ANCOVA.

Correlations	Groups	Species	Treatment	r^2	*P*	Group slope (k) 95% CI	Common slope (k) 95% CI	Elevation (b) 95% CI	Test of common lines	
									P_k	P_b
A_m~SLA	angiosperm	13	control	0.40	**0.02**	1.12 (0.68, 1.83)	1.06 (0.76, 1.47)	0.05 (-0.78, 0.88)	0.75	0.82
			warming	0.48	**<0.01**	1.01 (0.64, 1.61)		0.04 (-0.80, 0.87)		
	gymnosperm	13	control	0.62	**<0.01**	2.55 (1.71, 3.80)	2.49 (1.85, 3.36)	-3.32 (-5.00, -1.65)	0.85	0.92
			warming	0.43	**0.02**	2.41 (1.49, 3.91)		-3.31 (-4.99, -1.63)		
A_m~N_m	angiosperm	8	control	0.30	0.16					
			warming	0.30	0.16					
	gymnosperm	4	control	-	-					
			warming	-	-					
N_m~SLA	angiosperm	19	control	0.17	0.08					
			warming	0.16	0.10					
	gymnosperm	11	control	0.01	0.74					
			warming	0.01	0.73					
A_m~SLA	angiosperm	37	control	0.79	**<0.001**	1.23 (1.06, 1.44)	1.35 (1.18, 1.54)	-0.69 (-1.07, -0.33)	0.06	**<0.001**
			drought	0.58	**<0.001**	1.61 (1.30, 2.01)		-0.85 (-1.22, -0.48)		
	gymnosperm	2	control	-	-					
			drought	-	-					
A_m~N_m	angiosperm	25	control	0.17	**0.04**	2.48(1.69, 3.64)	2.74 (2.10, 3.56)	1.46 (1.22, 1.70)	0.48	0.17
			drought	0.26	**0.01**	2.99 (2.08, 4.30)		1.32 (1.09, 1.56)		
	gymnosperm	0	control	-	-					
			drought	-	-					
N_m~SLA	angiosperm	39	control	0.05	0.93					
			drought	0.04	0.24					

	gymnosperm	0	control	-	-					
			drought	-	-					
A_m~SLA	angiosperm	61	control	0.27	**<0.001**	1.46 (1.17, 1.82)	1.48 (1.26, 1.72)	-1.08 (-1.61, -0.56)	0.89	**<0.01**
			eCO_2	0.28	**<0.001**	1.49 (1.20, 1.86)		-0.93 (-1.44, -0.42)		
	gymnosperm	27	control	0.28	**<0.01**	1.81 (1.29, 2.55)	1.91 (1.51, 2.41)	-1.91 (-2.81, -1.02)	0.66	0.36
			eCO_2	0.36	**<0.001**	2.01 (1.45, 2.77)		-1.82 (-2.70, -0.94)		
A_m~N_m	angiosperm	44	control	0.19	**<0.01**	1.96 (1.49, 2.59)	1.84 (1.50, 2.24)	1.63 (1.48, 1.78)	0.49	**<0.01**
			eCO_2	0.14	**0.01**	1.71 (1.28, 2.27)		1.83 (1.70, 1.97)		
	gymnosperm	18	control	0.002	0.87					
			eCO_2	0.001	0.95					
N_m~SLA	angiosperm	94	control	0.19	**<0.001**	0.89 (0.74, 1.07)	0.93 (0.81, 1.06)	-1.74 (-2.03, -1.46)	0.48	0.43
			eCO_2	0.13	**<0.001**	0.97 (0.80, 1.18)		-1.77 (-2.04, -1.49)		
	gymnosperm	23	control	0.50	**<0.001**	0.80 (0.59, 1.10)	0.83 (0.67, 1.05)	-1.41 (-1.80, -1.03)	0.72	0.55
			eCO_2	0.45	**<0.001**	0.63 (1.21, 0.83)		-1.44 (-1.82, -1.06)		
A_m~SLA	angiosperm	48	control	0.32	**<0.001**	1.05 (0.83, 1.34)	1.08 (0.92, 1.27)	-0.24 (-0.61, 0..13)	0.76	0.43
			nitrogen	0.49	**<0.001**	1.10 (0.89, 1.36)		-0.20 (-0.57, 0.17)		
	gymnosperm	12	control	0.13	0.25					
			nitrogen	0.15	0.21					
A_m~N_m	angiosperm	43	control	0.38	**<0.001**	1.59 (1.24, 2.04)	1.76 (1.48, 2.10)	1.65 (1.55, 1.76)	0.25	**<0.01**
			nitrogen	0.37	**<0.001**	1.95 (1.52, 2.50)		1.47 (1.35, 1.60)		
	gymnosperm	15	control	0.28	**0.04**	1.29 (0.79, 2.10)	1.42 (1.03, 1.93)	1.51 (1.34, 1.68)	0.60	0.37
			nitrogen	0.50	**<0.01**	1.51 (1.00, 2.29)		1.42 (1.25, 1.59)		
N_m~SLA	angiosperm	59	control	0.49	**<0.001**	0.81 (0.67, 0.88)	0.77 (0.67, 0.88)	-1.42 (-1.64, -1.20)	0.41	**<0.001**
			nitrogen	0.49	**<0.001**	0.73 (0.60, 0.94)		-1.29 (-1.51, -1.08)		
	gymnosperm	15	control	0.33	**0.02**	1.03 (0.64, 1.65)	1.03 (0.74, 1.43)	-2.00 (-2.72, -1.28)	0.98	0.08
			nitrogen	0.31	**0.03**	1.03 (0.64, 1.65)		-1.84 (-2.55, -1.13)		

Values represent the coefficient of determination (r^2), statistical significance (P), slope (k) and elevation (b) of the trait relationships, and the statistical significance of the effect of global environmental changes on the slope (P_k) or elevation (P_b) of trait relationships.

Supplementary Table 7. Analysis of SMA regression values for trait relationships (of the form $\log y = k * \log x + b$) under control and treatment conditions (warming, eCO_2, drought and nitrogen) between dicotyledons and monocotyledons. The homogeneity among SMA slopes via a permutation test and for differences in SMA elevation via the SMA analogue of standard ANCOVA.

Correlations	Groups	Species	Treatment	r^2	*P*	Group slope (k) 95% CI	Common slope (k) 95% CI	Elevation (b) 95% CI	Test of common lines	
									P_k	P_b
A_m~SLA	dicotyledon	23	control	0.37	**<0.01**	1.18 (0.83, 1.68)	1.21 (0.94, 1.55)	-0.33 (-1.05, 0.39)	0.85	0.78
			warming	0.33	**<0.01**	1.24 (0.86, 1.68)		-0.35 (-1.07, 0.38)		
	monocotyledon	6	control	0.23	0.33					
			warming	0.06	0.64					
A_m~N_m	dicotyledon	10	control	0.38	0.06					
			warming	0.30	0.10					
	monocotyledon	1	control	-	-					
			warming	-	-					
N_m~SLA	dicotyledon	23	control	0.20	**0.03**					
			warming	0.16	0.06					
	monocotyledon	1	control	-	-					
			warming	-	-					
A_m~SLA	dicotyledon	41	control	0.74	**<0.001**	1.16 (0.98, 1.36)	1.26 (1.10, 1.45)	-0.53 (-0.89, -0.17)	0.07	**<0.001**
			drought	0.54	**<0.001**	1.48 (1.19, 1.84)		-0.67 (-1.02, -0.31)		
	monocotyledon	11	control	0.68	**<0.01**	1.26 (0.83, 1.90)	1.19 (0.94, 1.52)	-0.11 (-0.76, 0.54)	0.73	0.36
			drought	0.83	**<0.001**	1.16 (0.85, 1.57)		-0.15 (-0.76, 0.46)		
A_m~N_m	dicotyledon	26	control	0.17	**0.04**	2.47(1.70, 3.59)	2.68 (2.07, 3.47)	1.48 (1.25, 1.70)	0.54	0.11
			drought	0.26	**<0.01**	2.89 (2.02, 4.13)		1.33 (1.10, 1.55)		
	monocotyledon	0	control	-	-					
			drought	-	-					
N_m~SLA	dicotyledon	43	control	0.07	0.08					
			drought	0.07	0.08					
	monocotyledon	12	control	0.39	0.03	1.38 (0.82, 2.34)	1.26 (0.88, 1.82)	-2.70 (-3.83, -1.56)	0.62	0.51

			drought	0.41	0.02	1.16 (0.69, 1.94)		-2.64 (-3.79, -1.49)		
A_m~SLA	dicotyledon	83	control	0.30	**<0.001**	1.53 (1.27, 1.84)	1.55 (1.36, 1.77)	-1.23 (-1.70, -0.76)	0.85	**<0.01**
			eCO_2	0.28	**<0.001**	1.57 (1.30, 1.89)		-1.08 (-1.53, -0.62)		
	monocotyledon	27	control	0.13	0.06					
			eCO_2	0.25	**<0.01**					
A_m~N_m	dicotyledon	63	control	0.25	**<0.001**	1.54 (1.24, 1.92)	1.45 (1.24, 1.70)	1.74 (1.63, 1.86)	0.44	**<0.01**
			eCO_2	0.20	**<0.001**	1.36 (1.08, 1.71)		1.91 (1.81, 2.01)		
	monocotyledon	25	control	0.13	0.08					
			eCO_2	0.21	**0.02**					
N_m~SLA	dicotyledon	122	control	0.30	**<0.001**	0.99 (0.85, 1.15)	1.03 (0.92, 1.14)	-1.95 (-2.20, -1.69)	0.50	0.50
			eCO_2	0.22	**<0.001**	1.07 (0.91, 1.25)		-1.97 (-2.22, -1.72)		
	monocotyledon	29	control	0.25	**<0.01**	1.41 (1.01, 1.98)	1.42 (1.12, 1.79)	-2.89 (-3.67, -2.10)	0.99	0.68
			eCO_2	0.25	**<0.01**	1.42 (1.01, 1.98)		-2.91 (-3.69, -2.13)		
A_m~SLA	dicotyledon	82	control	0.34	**<0.001**	1.26 (1.05, 1.51)	1.30 (1.15, 1.46)	-0.69 (-1.04, -0.34)	0.66	0.19
			nitrogen	0.44	**<0.001**	1.33 (1.13, 1.57)		-0.64 (-0.99, -0.30)		
	monocotyledon	24	control	0.52	**<0.001**	1.29 (0.96, 1.75)	1.32 (1.08, 1.63)	-0.74 (-1.38, -0.11)	0.82	0.14
			nitrogen	0.56	**<0.001**	1.35 (1.01, 1.81)		-0.67 (-1.31, -0.04)		
A_m~N_m	dicotyledon	77	control	0.41	**<0.001**	1.45 (1.22, 1.73)	1.54 (1.37, 1.75)	1.77 (1.70, 1.84)	0.33	**<0.001**
			nitrogen	0.42	**<0.001**	1.64 (1.38, 1.96)		1.61 (1.53, 1.70)		
	monocotyledon	24	control	0.19	**0.03**					
			nitrogen	0.06	0.26					
N_m~SLA	dicotyledon	101	control	0.35	**<0.001**	0.91 (0.78, 1.07)	0.88 (0.79, 0.98)	-1.68 (-1.89, -1.47)	0.46	**<0.001**
			nitrogen	0.45	**<0.001**	0.84 (0.73, 0.98)		-1.55 (-1.75, -1.34)		
	monocotyledon	30	control	0.18	**0.02**	0.87 (0.62, 1.23)	0.81 (0.64, 1.03)	-1.61 (-2.05, -1.17)	0.57	**<0.001**
			nitrogen	0.25	**<0.01**	0.76 (0.55, 1.06)		-1.44 (-1.87, -1.00)		

Values represent the coefficient of determination (r^2), statistical significance (P), slope (k) and elevation (b) of the trait relationships, and the statistical significance of the effect of global environmental changes on the slope (P_k) or elevation (P_b) of trait relationships.

Supplementary Table 8. Analysis of SMA regression values for trait relationships (of the form $\log y = k * \log x + b$) under control and treatment conditions (warming, eCO_2, drought and nitrogen) between C_3 herb and C_4 herb. The homogeneity among SMA slopes via a permutation test and for differences in SMA elevation via the SMA analogue of standard ANCOVA.

Correlations	Groups	Species	Treatment	r^2	*P*	Group slope (k) 95% CI	Common slope (k) 95% CI	Elevation (b) 95% CI	Test of common lines P_k	P_b
A_m~SLA	C_3 herb	16	control	0.31	**0.03**					
			warming	0.18	0.09					
	C_4 herb	0	control	-	-					
			warming	-	-					
A_m~N_m	C_3 herb	3	control	-	-					
			warming	-	-					
	C_4 herb	0	control	-	-					
			warming	-	-					
N_m~SLA	C_3 herb	5	control	0.92	**0.01**					
			warming	0.68	0.08					
	C_4 herb	0	control	-	-					
			warming	-	-					
A_m~SLA	C_3 herb	4	control	-	-					
			drought	-	-					
	C_4 herb	11	control	0.68	**<0.01**	1.26 (0.83, 1.90)	1.19 (0.94, 1.52)	-0.11 (-0.76, 0.54)	0.73	0.36
			drought	0.83	**<0.001**	1.16 (0.85, 1.57)		-0.15 (-0.76, 0.46)		
A_m~N_m	C_3 herb	1	control	-	-					
			drought	-	-					
	C_4 herb	0	control	-	-					
			drought	-	-					
N_m~SLA	C_3 herb	7	control	0.36	0.15					
			drought	0.06	0.61					
	C_4 herb	9	control	0.40	0.07					

			drought	0.50	**0.03**					
A_m~SLA	C_3 herb	38	control	0.33	**<0.001**	2.14 (1.63, 2.81)	2.21 (1.82, 2.68)	-2.78 (-3.80, -1.75)	0.75	**0.03**
			eCO_2	0.30	**<0.001**	2.28 (1.72, 3.01)		-2.60 (-3.59, -1.61)		
	C_4 herb	10	control	0.16	0.26					
			eCO_2	0.06	0.48					
A_m~N_m	C_3 herb	34	control	0.24	**<0.01**	1.46 (1.07, 1.99)	1.40 (1.13, 1.74)	1.70 (1.53, 1.88)	0.69	**0.04**
			eCO_2	0.23	**<0.01**	1.34 (0.98, 1.83)		1.85 (1.68, 2.01)		
	C_4 herb	9	control	0.68	**<0.01**	1.21 (0.74, 1.97)	1.13 (0.80, 1.58)	2.18 (2.02, 2.34)	0.64	0.77
			eCO_2	0.64	**<0.01**	1.04 (0.62, 1.73)		2.20 (2.04, 2.36)		
N_m~SLA	C_3 herb	42	control	0.52	**<0.001**	1.22 (0.98, 1.52)	1.27 (1.09, 1.49)	-2.48 (-2.94, -2.01)	0.61	0.86
			eCO_2	0.48	**<0.001**	1.32 (1.05, 1.66)		-2.48 (-2.94, -2.03)		
	C_4 herb	14	control	0.34	**0.03**	2.12 (1.30, 3.47)	1.98 (1.41, 2.80)	-4.23 (-5.87, -2.59)	0.69	0.37
			eCO_2	0.32	**0.03**	1.85 (1.13, 3.05)		-4.29 (-5.95, -2.64)		
A_m~SLA	C_3 herb	49	control	0.31	**<0.001**	1.58(1.24, 2.01)	1.57 (1.32, 1.85)	-1.32 (-1.92, -0.72)	0.92	0.09
			nitrogen	0.35	**<0.001**	1.55 (1.23, 1.96)		-1.24 (-1.83, -0.65)		
	C_4 herb	7	control	0.68	**0.02**					
			nitrogen	0.56	0.05					
A_m~N_m	C_3 herb	49	control	0.50	**<0.001**	1.10 (0.89, 1.35)	1.17 (1.01, 1.36)	1.95 (1.88, 2.02)	0.38	**<0.001**
			nitrogen	0.51	**<0.001**	1.25 (1.02, 1.53)		1.80 (1.72, 1.88)		
	C_4 herb	7	control	0.01	0.86	-				
			nitrogen	0.001	0.94	-				
N_m~SLA	C_3 herb	63	control	0.36	**<0.001**	0.93 (0.81, 1.06)	0.89 (0.82, 0.98)	-1.72 (-1.90, -1.55)	0.47	**<0.001**
			nitrogen	0.45	**<0.001**	0.87 (0.76, 0.98)		-1.58 (-1.76, -1.40)		
	C_4 herb	7	control	0.003	0.91					
			nitrogen	0.15	0.40					

Values represent the coefficient of determination (r^2), statistical significance (P), slope (k) and elevation (b) of the trait relationships, and the statistical significance of the effect of global environmental changes on the slope (P_k) or elevation (P_b) of trait relationships.

Supplementary Table 9. Analysis of SMA regression values for trait relationships (of the form $\log y = k * \log x + b$) under control and treatment conditions (warming, eCO_2, drought and nitrogen) between field and environmentally controlled experiments. The homogeneity among SMA slopes via a permutation test and for differences in SMA elevation via the SMA analogue of standard ANCOVA.

Correlations	Groups	Species	Treatment	r^2	P	Group slope (k) 95% CI	Common slope (k) 95% CI	Elevation (b) 95% CI	Test of common lines	
									P_k	P_b
A_m~SLA	field	12	control	0.52	**<0.01**	1.38 (0.86, 2.20)	1.41 (1.00, 2.00)	-1.07 (-2.19, 0.05)	0.86	0.68
			warming	0.34	**0.04**	1.46 (0.85, 2.52)		-1.02 (-2.15, 0.11)		
	controlled	30	control	0.39	**<0.001**	1.97 (1.46, 2.65)	1.95 (1.58, 2.41)	-2.06 (-3.03, -1.09)	0.93	0.51
			warming	0.33	**<0.001**	1.93 (1.42, 2.64)		-2.11 (-3.10, -1.13)		
A_m~N_m	field	2	control	-	-					
			warming	-	-					
	controlled	13	control	0.49	**<0.01**	1.96 (1.24, 3.10)	1.98 (1.43, 2.73)	1.62 (1.34, 1.90)	0.95	0.78
			warming	0.43	**0.02**	2.00 (1.23, 3.24)		1.65 (1.39, 1.92)		
N_m~SLA	field	14	control	0.24	0.08					
			warming	0.06	0.39					
	controlled	21	control	0.15	0.08					
			warming	0.08	0.21					
A_m~SLA	field	11	control	0.78	**<0.001**	1.37 (0.97, 1.94)	1.38 (1.13, 1.69)	-0.73 (-1.31, -0.14)	0.93	0.12
			drought	0.87	**<0.001**	1.39 (1.07, 1.82)		-0.85 (-1.43, -0.28)		
	controlled	43	control	0.53	**<0.001**	1.28 (1.03, 1.58)	1.45 (1.23, 1.73)	-0.88 (-1.41, -0.35)	0.06	**<0.05**
			drought	0.30	**<0.001**	1.76 (1.35, 2.28)		-0.99 (-1.51, -0.47)		
A_m~N_m	field	8	control	0.38	0.10					
			drought	0.56	**0.03**					
	controlled	18	control	0.31	**0.02**					
			drought	0.23	0.05					
N_m~SLA	field	11	control	0.67	**<0.01**	0.42 (0.28, 0.64)	0.38 (0.29, 0.50)	-0.49 (-0.72, -0.25)	0.51	0.46
			drought	0.73	**<0.001**	0.35 (0.24, 0.52)		-0.51 (-0.74, -0.28)		

	controlled	44	control	0.07	0.08					
			drought	0.08	0.06					
A_m~SLA	field	57	control	0.30	**<0.001**	1.51 (1.21, 1.90)	1.50 (1.29, 1.75)	-1.09 (-1.06, -0.59)	0.93	**0.02**
			eCO_2	0.38	**<0.001**	1.49 (1.21, 1.84)		-0.96 (-1.46, -0.47)		
	controlled	80	control	0.49	**<0.001**	1.78 (1.51, 2.09)	1.85 (1.65, 2.07)	-1.92 (-2.40, -1.44)	0.50	**0.01**
			eCO_2	0.50	**<0.001**	1.92 (1.64, 2.25)		-1.78 (-2.25, -1.32)		
A_m~N_m	field	52	control	0.13	**<0.01**	2.34 (1.80, 3.04)	2.12 (1.80, 2.62)	1.59 (1.45, 1.73)	0.42	**0.01**
			eCO_2	0.11	**0.02**	2.01 (1.5, 2.63)		1.78 (1.65, 1.90)		
	controlled	54	control	0.19	**<0.01**	1.45 (1.13, 1.85)	1.38 (1.16, 1.65)	1.73 (1.59, 1.86)	0.62	**0.03**
			eCO_2	0.22	**<0.001**	1.33 (1.04, 1.69)		1.87 (1.75, 1.99)		
N_m~SLA	field	83	control	0.21	**<0.001**	0.76 (0.62, 0.92)	0.80 (0.70, 0.92)	-1.47 (-1.72, -1.22)	0.43	0.33
			eCO_2	0.17	**<0.001**	0.85 (0.69, 1.03)		-1.50 (-1.74, -1.25)		
	controlled	91	control	0.37	**<0.001**	1.01 (0.86, 1.20)	1.04 (0.92, 1.17)	-1.94 (-2.23, -1.65)	0.69	0.44
			eCO_2	0.27	**<0.001**	1.06 (0.89, 1.27)		-1.97 (-2.25, -1.68)		
A_m~SLA	field	40	control	0.36	**<0.001**	1.48 (1.14, 1.92)	1.44 (1.20, 1.72)	-1.01 (-1.59, -0.44)	0.78	0.44
			nitrogen	0.41	**<0.001**	1.40 (1.10, 1.80)		-0.97 (-1.54, -0.40)		
	controlled	78	control	0.46	**<0.001**	1.18 (0.99, 1.39)	1.23 (1.10, 1.37)	-0.54 (-0.84, -0.23)	0.50	0.07
			nitrogen	0.54	**<0.001**	1.27 (1.09, 1.48)		-0.47 (-0.77, -0.17)		
A_m~N_m	field	39	control	0.37	**<0.001**	1.94 (1.49, 2.52)	1.87 (1.56, 2.27)	1.58 (1.45, 1.71)	0.77	0.25
			nitrogen	0.32	**<0.001**	1.83 (1.40, 2.40)		1.51 (1.36, 1.66)		
	controlled	77	control	0.32	**<0.001**	1.58 (1.31, 1.91)	1.70 (1.49, 1.94)	1.81 (1.73, 1.89)	0.31	**<0.001**
			nitrogen	0.38	**<0.001**	1.81 (1.51, 2.17)		1.56 (1.47, 1.66)		
N_m~SLA	field	57	control	0.38	**<0.001**	0.76 (0.61,0.94)	0.73 (0.63, 0.85)	-1.29 (-1.54, -1.05)	0.63	**0.02**
			nitrogen	0.70	**<0.001**	0.70 (0.56, 0.88)		-1.23 (-1.47, -0.98)		
	controlled	89	control	0.41	**<0.001**	0.93 (0.79, 1.10)	0.92 (0.82, 1.02)	-1.84 (-2.06, -1.62)	0.80	**<0.001**
			nitrogen	0.50	**<0.001**	0.90 (0.78, 1.05)		-1.65 (-1.87, -1.43)		

Values represent the coefficient of determination (r^2), statistical significance (P), slope (k) and elevation (b) of the trait relationships, and the statistical significance of the effect of global environmental changes on the slope (P_k) or elevation (P_b) of trait relationships.

Supplementary Table 10. Analysis of SMA regression values for trait relationships (of the form $\log y = k * \log x + b$) under control and treatment conditions (warming, eCO_2, drought and nitrogen) between low strength and high strength. The homogeneity among SMA slopes via a permutation test and for differences in SMA elevation via the SMA analogue of standard ANCOVA.

Correlations	Groups	Species	Treatment	r^2	*P*	Group slope (k) 95% CI	Common slope (k) 95% CI	Elevation (b) 95% CI	Test of common lines	
									P_k	P_b
A_m~SLA	low strength	20	control	0.56	**<0.001**	2.04 (1.48, 2.83)	2.09 (1.65, 2.64)	-2.45 (-3.54, -1.37)	0.84	0.72
			warming	0.49	**<0.001**	2.14 (1.51, 3.03)		-2.42 (-3.51, -1.33)		
	high strength	22	control	0.23	**0.02**					
			warming	0.14	0.09					
A_m~N_m	low strength	8	control	0.39	0.10					
			warming	0.30	0.16					
	high strength	7	control	0.68	**0.02**	1.94 (1.05, 3.58)	2.05 (1.36, 3.10)	1.62 (1.22, 2.03)	0.75	0.97
			warming	0.67	**0.03**	2.19 (1.17, 4.08)		1.62 (1.25, 1.98)		
N_m~SLA	low strength	17	control	0.09	0.23					
			warming	0.11	0.20					
	high strength	18	control	0.24	0.04					
			warming	0.07	0.28					
A_m~SLA	low strength	39	control	0.73	**<0.001**	1.39 (1.17, 1.65)	1.39 (1.23, 1.57)	-0.71 (-1.07, -0.34)	0.99	0.31
			drought	0.71	**<0.001**	1.39 (1.16, 1.66)		-0.75 (-1.11, -0.39)		
	high strength	15	control	0.60	**<0.001**	1.05 (0.72, 1.52)	1.25 (0.93, 1.70)	-0.54 (-1.33, 0.26)	0.13	**<0.001**
			drought	0.43	**<0.01**	1.61 (1.04, 2.49)		-0.86 (-1.64, -0.08)		
A_m~N_m	low strength	20	control	0.08	0.22					
			drought	0.06	0.29					
	high strength	6	control	0.76	**0.02**					
			drought	0.60	0.07					
N_m~SLA	low strength	31	control	0.02	0.43					
			drought	0.05	0.22					

	high strength	24	control	0.35	**<0.01**	0.87 (0.61, 1.23)	0.77 (0.60, 0.98)	-1.51 (-1.93, -1.08)	0.34	0.30
			drought	0.43	**<0.001**	0.69 (0.50, 0.96)		-1.47 (-1.89, -1.04)		
A_m~SLA	low strength	53	control	0.42	**<0.001**	1.64 (1.33, 2.03)	1.63 (1.40, 1.90)	-1.42 (-1.97, -0.86)	0.94	**0.03**
			eCO_2	0.35	**<0.001**	1.62 (1.30, 2.03)		-1.29 (-1.83, -0.74)		
	high strength	84	control	0.42	**<0.001**	1.65 (1.40, 1.95)	1.73 (1.54, 1.93)	-1.61 (-2.06, -1.17)	0.49	**0.01**
			eCO_2	0.49	**<0.001**	1.79 (1.53, 2.10)		-1.48 (-1.91, -1.05)		
A_m~N_m	low strength	53	control	0.34	**<0.001**	1.92 (1.53, 2.41)	1.92 (1.63, 2.26)	1.49 (1.36, 1.62)	0.98	**<0.01**
			eCO_2	0.28	**<0.001**	1.91 (1.51, 2.42)		1.68 (1.56, 1.79)		
	high strength	53	control	0.16	**<0.01**	1.42 (1.10, 1.83)	1.33 (1.11, 1.59)	1.85 (1.73. 1.98)	0.49	**0.04**
			eCO_2	0.20	**<0.001**	1.25 (0.98, 1.61)		1.98 (1.87, 2.10)		
N_m~SLA	low strength	77	control	0.41	**<0.001**	0.92 (0.77, 1.09)	0.91 (0.80, 1.03)	-1.65 (-1.91, -1.39)	0.81	0.32
			eCO_2	0.32	**<0.001**	0.89 (0.74, 1.08)		-1.68 (-1.93, -1.42)		
	high strength	97	control	0.28	**<0.001**	0.94 (0.79, 1.11)	0.99 (0.88, 1.12)	-1.88 (-2.16, -1.60)	0.35	0.47
			eCO_2	0.24	**<0.001**	1.05 (0.88, 1.26)		-1.91 (-2.18, -1.63)		
A_m~SLA	low strength (mmol L^{-1})	38	control	0.45	**<0.001**	1.04 (0.81, 1.33)	1.08 (0.91, 1.28)	-0.17 (-0.56, 0.22)	0.70	0.31
			nitrogen	0.52	**<0.001**	1.11 (0.88, 1.40)		-0.11 (-0.50, 0.27)		
	high strength (mmol L^{-1})	27	control	0.30	**<0.01**	1.48 (1.06, 2.07)	1.50 (1.21, 1.86)	-1.24 (-2.01, -0.48)	0.91	0.16
			nitrogen	0.50	**<0.001**	1.52 (1.14, 2.03)		-1.15 (-1.90, -0.40)		
	low strength (kgN ha^{-1} yr^{-1})	18	control	0.46	**<0.01**	1.58 (1.08, 2.31)	1.56 (1.21, 2.00)	-1.19 (-2.08, -0.30)	0.91	0.72
			nitrogen	0.54	**<0.001**	1.54 (1.08, 2.18)		-1.16 (-2.05, -0.27)		
	high strength (kgN ha^{-1} yr^{-1})	15	control	0.24	0.06					
			nitrogen	0.32	**0.03**					
A_m~N_m	low strength (mmol L^{-1})	42	control	0.36	**<0.001**	1.86 (1.45, 2.40)	2.05 (1.73, 2.44)	1.68 (1.56, 1.80)	0.29	**<0.001**
			nitrogen	0.46	**<0.001**	2.23 (1.77, 2.82)		1.35 (1.20, 1.49)		
	high strength (mmol L^{-1})	27	control	0.54	**<0.001**	1.17 (0.89, 1.54)	1.23 (1.02, 1.48)	1.97 (1.88, 2.05)	0.60	**<0.01**
			nitrogen	0.61	**<0.001**	1.29 (1.00, 1.66)		1.80 (1.69, 1.90)		
	low strength (kgN ha^{-1} yr^{-1})	17	control	0.22	0.06					
			nitrogen	0.10	0.21					

	high strength (kgN ha^{-1} yr^{-1})	14	control	0.79	<**0.001**	1.61 (1.21, 2.14)	1.54 (1.27, 1.86)	1.56 (1.43, 1.69)	0.62	0.50
			nitrogen	0.81	<**0.001**	1.47 (1.12, 1.93)		1.52 (1.38, 1.67)		
N_m~SLA	low strength (mmol L^{-1})	35	control	0.37	<**0.001**	0.78 (0.59, 1.03)	0.69 (0.57, 0.84)	-1.31 (-1.59, -1.02)	0.24	<**0.001**
			nitrogen	0.45	<**0.001**	0.62 (0.48, 0.81)		-1.10 (-1.39, -0.82)		
	high strength (mmol L^{-1})	32	control	0.48	<**0.001**	1.16 (0.89, 1.52)	1.13 (0.96, 1.33)	-2.40 (-2.83, -1.98)	0.81	<**0.001**
			nitrogen	0.69	<**0.001**	1.12 (0.91, 1.37)		-2.19 (-2.61, -1.77)		
	low strength (kgN ha^{-1} yr^{-1})	19	control	0.05	0.36					
			nitrogen	0.03	0.45					
	high strength (kgN ha^{-1} yr^{-1})	32	control	0.50	<**0.001**	0.76 (0.59, 0.99)	0.75 (0.62, 0.90)	-1.30 (-1.61, -1.00)	0.86	0.08
			nitrogen	0.46	<**0.001**	0.73 (0.56, 0.96)		-1.24 (-1.54, -0.94)		

Values represent the coefficient of determination (r^2), statistical significance (P), slope (k) and elevation (b) of the trait relationships, and the statistical significance of the effect of global environmental changes on the slope (P_k) or elevation (P_b) of trait relationships.

Supplementary Table 11. Analysis of SMA regression values for trait relationships (of the form $\log y = k * \log x + b$) under control and treatment conditions (warming, eCO_2, drought and nitrogen) between with and without species overlap. The homogeneity among SMA slopes via a permutation test and for differences in SMA elevation via the SMA analogue of standard ANCOVA.

Correlations	Groups	Species	Treatment	r^2	P	Group slope (k) 95% CI	Common slope (k) 95% CI	Elevation (b) 95% CI	Test of common lines	
									P_k	P_b
A_m~SLA	with overlap	42	control	0.48	**<0.001**	1.92 (1.53, 2.41)	1.88 (1.59, 2.22)	-1.95 (-2.65, -1.24)	0.80	0.73
			warming	0.42	**<0.001**	1.84 (1.44, 2.34)		-1.97 (-2.68, -1.25)		
	without overlap	38	control	0.43	**<0.001**	1.85 (1.44, 2.38)	1.82 (1.52, 2.18)	-1.80 (-2.56, -1.04)	0.83	0.77
			warming	0.38	**<0.001**	1.78 (1.37, 2.32)		-1.82 (-2.58, -1.05)		
A_m~N_m	with overlap	15	control	0.55	**<0.01**	1.90 (1.28, 2.82)	1.91 (1.44, 2.54)	1.64 (1.42, 1.86)	0.95	0.80
			warming	0.46	**<0.01**	1.93 (1.26, 2.96)		1.66 (1.45, 1.880		
	without overlap	13	control	0.51	**<0.01**	1.73 (1.10, 2.71)	1.78 (1.29, 2.46)	1.68 (1.43, 1.92)	0.86	0.79
			warming	0.40	**0.02**	1.83 (1.12, 3.01)		1.70 (1.46, 1.94)		
N_m~SLA	with overlap	35	control	0.16	**0.02**					
			warming	0.08	0.10					
	without overlap	29	control	0.14	0.05					
			warming	0.07	0.16					
A_m~SLA	with overlap	54	control	0.67	**<0.001**	1.32 (1.12, 1.54)	1.42 (1.26, 1.62)	-0.81 (-1.19, -0.43)	0.13	**0.01**
			drought	0.48	**<0.001**	1.61 (1.32, 1.96)		-0.93 (-1.30, -0.56)		
	without overlap	48	control	0.68	**<0.001**	1.37 (1.16, 1.62)	1.45 (1.27, 1.65)	-0.86 (-1.27, -0.45)	0.28	**0.03**
			drought	0.47	**<0.001**	1.59 (1.28, 1.97)		-0.97 (-1.37, -0.57)		
A_m~N_m	with overlap	26	control	0.17	**0.04**	2.47 (1.70, 3.59)	2.68 (2.07, 3.47)	1.48 (1.25, 1.70)	0.54	0.11
			drought	0.25	**<0.01**	2.89 (2.02, 4.13)		1.33 (1.10, 1.55)		
	without overlap	25	control	0.18	**0.03**	2.57 (1.75, 3.76)	2.62 (2.01, 3.41)	1.49 (1.27, 1.72)	0.88	0.13
			drought	0.21	**0.02**	2.67 (1.84, 3.88)		1.36 (1.13, 1.59)		
N_m~SLA	with overlap	55	control	0.09	**0.02**	0.75 (0.58, 0.97)	0.75 (0.63, 0.90)	-1.40 (-1.72, -1.09)	0.99	0.39
			drought	0.13	**<0.01**	0.75 (0.58, 0.97)		-1.37 (-1.68, -1.06)		

	without overlap	52	control	0.09	**0.03**	0.79 (0.60, 1.03)	0.78 (0.64, 0.94)	-1.47 (-1.81, -1.14)	0.92	0.36
			drought	0.12	**0.01**	0.77 (0.59, 1.00)		-1.44 (-1.77, -1.10)		
A_m~SLA	with overlap	137	control	0.43	**<0.001**	1.65 (1.45, 1.88)	1.70 (1.55, 1.86)	-1.56 (-1.90, -1.21)	0.57	**<0.001**
			eCO_2	0.45	**<0.001**	1.74 (1.54, 1.98)		-1.42 (-1.76, -1.09)		
	without overlap	116	control	0.46	**<0.001**	1.64 (1.43, 1.87)	1.68 (1.53, 1.85)	-1.54 (-1.90, -1.18)	0.59	**<0.001**
			eCO_2	0.48	**<0.001**	1.72 (1.51, 1.97)		-1.40 (-1.75, -1.05)		
A_m~N_m	with overlap	106	control	0.21	**<0.001**	1.67 (1.40, 1.98)	1.58 (1.40, 1.79)	1.69 (1.60, 1.78)	0.39	**<0.01**
			eCO_2	0.20	**<0.001**	1.50 (1.26, 1.78)		1.84 (1.76, 1.93)		
	without overlap	87	control	0.23	**<0.001**	1.64 (1.36, 1.98)	1.52 (1.33, 1.74)	1.69 (1.59, 1.79)	0.25	**<0.01**
			eCO_2	0.23	**<0.001**	1.41 (1.17, 1.70)		1.84 (1.75, 1.93)		
N_m~SLA	with overlap	174	control	0.32	**<0.001**	0.92 (0.82, 1.05)	0.94 (0.85, 1.03)	-1.75 (-1.95, -1.55)	0.69	0.96
			eCO_2	0.31	**<0.001**	0.96 (0.83, 1.11)		-1.75 (-1.94, -1.55)		
	without overlap	135	control	0.29	**<0.001**	0.93 (0.81, 1.08)	0.97 (0.88, 1.08)	-1.82 (-2.05, -1.60)	0.45	0.35
			eCO_2	0.25	**<0.001**	1.01 (0.87, 1.17)		-1.84 (-2.06, -1.62)		
A_m~SLA	with overlap	118	control	0.42	**<0.001**	1.26 (1.09, 1.44)	1.28 (1.17, 1.41)	-0.66 (-0.93, -0.40)	0.68	0.06
			nitrogen	0.50	**<0.001**	1.31 (1.15, 1.49)		-0.60 (-0.87, -0.34)		
	without overlap	109	control	0.42	**<0.001**	1.23 (1.06, 1.43)	1.25 (1.14, 1.39)	-0.59 (-0.87, -0.32)	0.74	0.08
			nitrogen	0.48	**<0.001**	1.28 (1.11, 1.46)		-0.54 (-0.81, -0.26)		
A_m~N_m	with overlap	116	control	0.32	**<0.001**	1.60 (1.37, 1.86)	1.71 (1.53, 1.90)	1.75 (1.68, 1.81)	0.23	**<0.001**
			nitrogen	0.36	**<0.001**	1.82 (1.57, 2.10)		1.56 (1.49, 1.64)		
	without overlap	109	control	0.32	**<0.001**	1.61 (1.38, 1.89)	1.72 (1.54, 1.92)	1.74 (1.67, 1.81)	0.25	**<0.001**
			nitrogen	0.34	**<0.001**	1.83 (1.57, 2.14)		1.56 (1.47, 1.64)		
N_m~SLA	with overlap	146	control	0.34	**<0.001**	0.92 (0.80, 1.05)	0.88 (0.80, 0.96)	-1.70 (-1.87, -1.53)	0.38	**<0.001**
			nitrogen	0.43	**<0.001**	0.85 (0.75, 0.96)		-1.56 (-1.73, -1.39)		
	without overlap	133	control	0.36	**<0.001**	0.93 (0.81, 1.06)	0.89 (0.81, 0.98)	-1.72 (-1.91, -1.54)	0.44	**<0.001**
			nitrogen	0.43	**<0.001**	0.86 (0.75, 0.98)		-1.58 (-1.76, -1.40)		

Values represent the coefficient of determination (r^2), statistical significance (P), slope (k) and elevation (b) of the trait relationships, and the statistical significance of the effect of global environmental changes on the slope (P_k) or elevation (P_b) of trait relationships.

Supplementary Notes

Supplementary Note 1: The consistency of major results between field and environmentally controlled experiments.

In this study, the experiments conducted in garden and field habitat were defined as field experiments. The experiments conducted in greenhouse, growth chamber and pot were classified as environmentally controlled experiments, in which the disturbances of the other variables were minimized. As a result, this study compiled a trait plasticity database from 102 field experiments and 108 environmentally controlled experiments (Figure 1a and Source Data). The consistency of major results between field and environmentally controlled experiments has been tested before using the whole dataset in global analyses. Considering the following results, we prefer to use the whole dataset in global analyses.

First, we have tested the trait relationships between field and environmentally controlled experiments, and found that trait relationships between different environmental types were mostly consistent (Figure 1). Only the A_m-N_m correlation from eCO_2 experiments showed different slopes (Figure 1h), which may partly result from the lower *R*-square of these two groups or the differences in species composition between environmental types. Overall, the results from environmentally controlled studies are consistent with that from the field experiments.

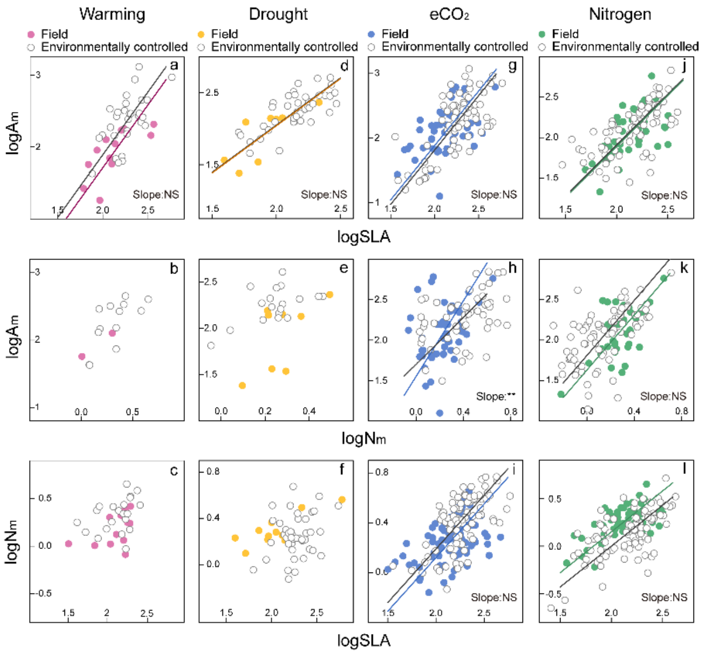

Figure 1. Comparison of trait relationships between field and environmentally controlled experiments. The homogeneity among SMA slopes via a permutation test. Significance: NS: $P > 0.05$; *$P < 0.05$; **$P < 0.01$; ***$P < 0.001$.

Second, we have tested the consistency between direction of trait plasticity and the intrinsic leaf trait relationships from different environmental types. The joint changes of leaf traits are divergent among species in both field and environmentally controlled experiments. Most of the trait plasticity were contrary to the LES (Figure 2). The consistent findings in both field and environmentally controlled experiments have strengthened our conclusion.

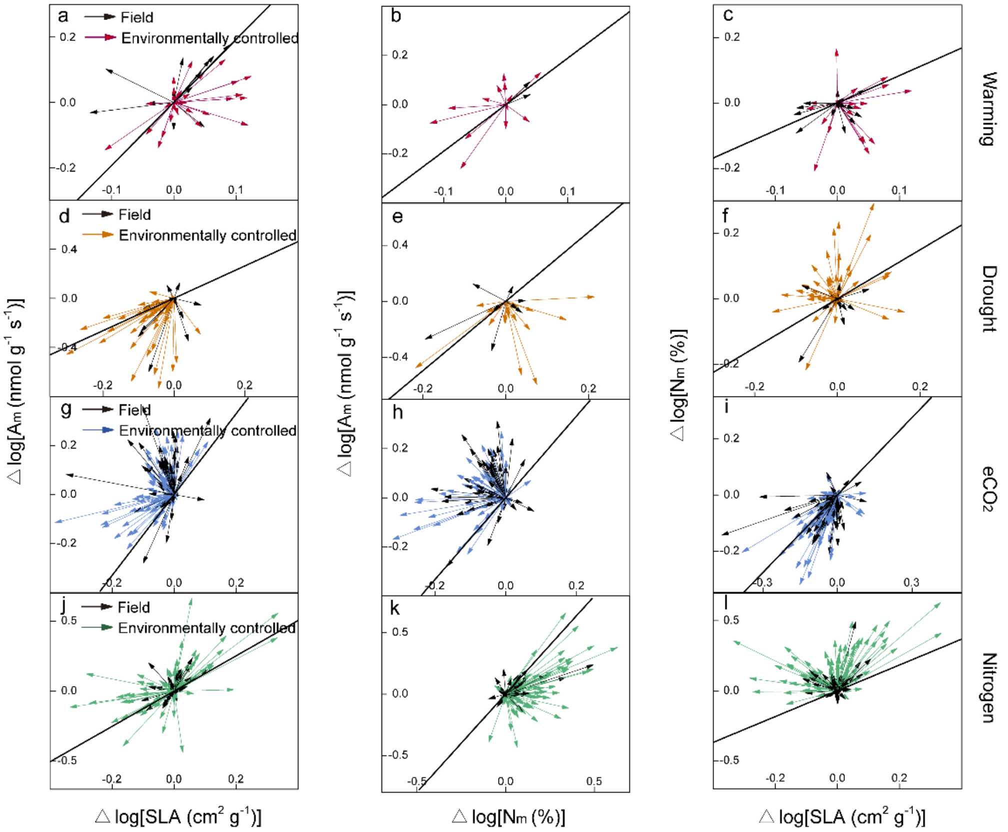

Figure 2. Plasticity of species-level pair-wise traits under global environmental changes. The black arrows represent field observations and the colored arrows represent environmentally controlled observations. Each colored arrow represents the direction of one species. The bold black line represents the intrinsic leaf trait relationship.

Supplementary Note 2: The presentation of species-level response into trait-trait space.

We plotted the variations of trait combinations induced by environmental changes for each species into trait-trait space. The variation of each species in a given trait could be quantified as the difference in trait values between control and treatment plots. Then the joint changes in pair-wise traits for each species could be represented by an arrow, the length of the arrow indicated the response size and the angle of the arrow characterized the direction of the joint changes. The starting point of each arrow was positioned at the origin (i.e., [0, 0]), and the final position was determined by the trait variations (i.e., [Δlog(trait*x*), Δlog(trait*y*)].
