# Supplementary material for: Robust leaf trait relationships across species under global environmental changes
Source: Nat Commun. 2020 Jun 12;11:2999. doi: 10.1038/s41467-020-16839-9 (PMC7293315; doi:10.1038/s41467-020-16839-9)
Supplement: Supplementary file 2 — Reporting Summary [file 41467_2020_16839_MOESM2_ESM.pdf]

nature research

Corresponding author(s): Jianyang Xia

Last updated by author(s): May 5, 2020

Reporting Summary

Nature Research wishes to improve the reproducibility of the work that we publish. This form provides structure for consistency and transparency in reporting. For further information on Nature Research policies, see Authors & Referees and the Editorial Policy Checklist .

Statistics

For all statistical analyses, confirm that the following items are present in the figure legend, table legend, main text, or Methods section.

n/a	Confirmed	
☐	☒	The exact sample size (*n*) for each experimental group/condition, given as a discrete number and unit of measurement
☐	☒	A statement on whether measurements were taken from distinct samples or whether the same sample was measured repeatedly
☐	☒	The statistical test(s) used AND whether they are one- or two-sided<br>*Only common tests should be described solely by name; describe more complex techniques in the Methods section.*
☒	☐	A description of all covariates tested
☐	☒	A description of any assumptions or corrections, such as tests of normality and adjustment for multiple comparisons
☐	☒	A full description of the statistical parameters including central tendency (e.g. means) or other basic estimates (e.g. regression coefficient) AND variation (e.g. standard deviation) or associated estimates of uncertainty (e.g. confidence intervals)
☐	☒	For null hypothesis testing, the test statistic (e.g. F, t, r) with confidence intervals, effect sizes, degrees of freedom and P value noted<br>*Give P values as exact values whenever suitable.*
☒	☐	For Bayesian analysis, information on the choice of priors and Markov chain Monte Carlo settings
☒	☐	For hierarchical and complex designs, identification of the appropriate level for tests and full reporting of outcomes
☐	☒	Estimates of effect sizes (e.g. Cohen's *d*, Pearson's *r*), indicating how they were calculated

Our web collection on statistics for biologists contains articles on many of the points above.

Software and code

Policy information about availability of computer code

Data collection	All original data were extracted from the text, tables, figures, and appendices of the publications. When data were graphically presented, GetData Graph Digitizer v2.26 was used to obtain numeric data (http://getdata-graph-digitizer.com/).
Data analysis	The DOS-based SMATR package in R version 3.6.1 was used for SMA regressions. The covariance error ellipse of covariant traits was drawn with Matlab 7.0. The response ratio of leaf traits was quantified with Metawin 2.0.

For manuscripts utilizing custom algorithms or software that are central to the research but not yet described in published literature, software must be made available to editors/reviewers. We strongly encourage code deposition in a community repository (e.g. GitHub). See the Nature Research guidelines for submitting code & software for further information.

Data

Policy information about availability of data

All manuscripts must include a data availability statement. This statement should provide the following information, where applicable:

- Accession codes, unique identifiers, or web links for publicly available datasets
- A list of figures that have associated raw data
- A description of any restrictions on data availability

We confirmed that if the manuscript was accepted, the data supporting the results will be archived in an appropriate public repository and the data DOI will be included at the end of the article.

Field-specific reporting

Please select the one below that is the best fit for your research. If you are not sure, read the appropriate sections before making your selection.

Ecological, evolutionary & environmental sciences study design

All studies must disclose on these points even when the disclosure is negative.

Study description	To quantify whether and how the trait relationships will shift under future global environmental changes, we examined the bivariate correlations between leaf net photosynthesis, leaf nitrogen content and specific leaf area in 210 experiments which mimic climate warming, drought, elevated CO2, and nitrogen deposition.
Research sample	We searched for peer-reviewed journal articles using ISI Web of Science and Google Scholar in March 2018 with no restriction on publication year. We focused on manipulative studies which included key global environmental changes: (1) warming, (2) eCO2, (3) drought, and (4) nitrogen addition. Drought here refers to rainfall reduction (field experiment) or irrigation reduction (environmentally controlled experiment). More than 4000 published articles reported changes of leaf traits (specific leaf area (SLA), leaf nitrogen content (N) and/or net photosynthetic rate (A)) under experimental manipulations. To minimize publication bias caused by different data sources, we only articles with at least two of the three leaf traits in this study.After systematic screening, a total of 415 published articles were included in our database, consisting of 520 species from 90 families.
Sampling strategy	We focused on manipulative studies which included leaf functional traits (leaf net photosynthesis, leaf nitrogen content and specific leaf area) and key global environmental changes (warming, eCO2, drought, and nitrogen addition). We collected leaf trait observations with mean, SD and sample size. The sample size of leaf trait was weighted when quantifying the effects size. The samples with incomplete statistics or experimental information were eliminated. This database contains all the manipulative studies that reported leaf traits, which provide enough data to estimate the effect of environmental changes.
Data collection	The data were collected from peer-reviewed journal articles by Erqian Cui. If the manuscript was accepted, the data supporting the results will be archived in an appropriate public repository
Timing and spatial scale	We searched for peer-reviewed journal articles using ISI Web of Science and Google Scholar when we start this research in March 2018 with no restriction on publication year and spatial scale.
Data exclusions	To minimize publication bias caused by different data sources, we considered only articles with at least two of the three leaf traits in this study.
Reproducibility	Our work was not an experiment but global analysis of published measurements. The selected measurements were all with effective replicates.
Randomization	This was not relevant to our study, because our work is not an experiment but a global analysis.
Blinding	Blinding was not relevant to our study because our data came from peer-reviewed journal articles.

Did the study involve field work? ☐ Yes ☒ No

Reporting for specific materials, systems and methods

We require information from authors about some types of materials, experimental systems and methods used in many studies. Here, indicate whether each material, system or method listed is relevant to your study. If you are not sure if a list item applies to your research, read the appropriate section before selecting a response.

Materials & experimental systems

n/a	Involved in the study
☒	☐ Antibodies
☒	☐ Eukaryotic cell lines
☒	☐ Palaeontology
☒	☐ Animals and other organisms
☒	☐ Human research participants
☒	☐ Clinical data

Methods

n/a	Involved in the study
☒	☐ ChIP-seq
☒	☐ Flow cytometry
☒	☐ MRI-based neuroimaging
